# Geographic Variation in Genomic Signals of Admixture Between Two Closely Related European Sepsid Fly Species

**DOI:** 10.1007/s11692-023-09612-5

**Published:** 2023-08-25

**Authors:** Athene Giesen, Wolf U. Blanckenhorn, Martin A. Schäfer, Kentaro K. Shimizu, Rie Shimizu-Inatsugi, Bernhard Misof, Lars Podsiadlowski, Oliver Niehuis, Heidi E. L. Lischer, Simon Aeschbacher, Martin Kapun

**Affiliations:** 1 Department of Evolutionary Biology and Environmental Studies, University of Zürich, Winterthurerstrasse 190, 8057 Zurich, Switzerland; 2 Zoological Research Museum Alexander Koenig, Bonn, Germany; 3 Department of Evolutionary Biology and Ecology, Institute of Biology I (Zoology), Albert Ludwig University, Freiburg, Germany; 4 Interfaculty Bioinformatics Unit, University of Bern, Bern, Switzerland; 5 Division of Cell & Developmental Biology, Medical University of Vienna, Vienna, Austria; 6 Natural History Museum Vienna, Burgring 7, 1010 Vienna, Austria

**Keywords:** ABBA–BABA test, Gene flow, Introgression, Reproductive isolation, Speciation, Sepsid flies

## Abstract

The extent of interspecific gene flow and its consequences for the initiation, maintenance, and breakdown of species barriers in natural systems remain poorly understood. Interspecific gene flow by hybridization may weaken adaptive divergence, but can be overcome by selection against hybrids, which may ultimately promote reinforcement. An informative step towards understanding the role of gene flow during speciation is to describe patterns of past gene flow among extant species. We investigate signals of admixture between allopatric and sympatric populations of the two closely related European dung fly species *Sepsis cynipsea* and *S. neocynipsea* (Diptera: Sepsidae). Based on microsatellite genotypes, we first inferred a baseline demographic history using Approximate Bayesian Computation. We then used genomic data from pooled DNA of natural and laboratory populations to test for past interspecific gene flow based on allelic configurations discordant with the inferred population tree (ABBA–BABA test with *D*-statistic). Comparing the detected signals of gene flow with the contemporary geographic relationship among interspecific pairs of populations (sympatric vs. allopatric), we made two contrasting observations. At one site in the French Cevennes, we detected an excess of past interspecific gene flow, while at two sites in Switzerland we observed lower signals of past microsatellite genotypes gene flow among populations in sympatry compared to allopatric populations. These results suggest that the species boundaries between these two species depend on the past and/or present eco-geographic context in Europe, which indicates that there is no uniform link between contemporary geographic proximity and past interspecific gene flow in natural populations.

**Supplementary Information:**

The online version contains supplementary material available at 10.1007/s11692-023-09612-5.

## Introduction

Speciation entails the evolution of reproductive isolation (RI) among lineages derived from a common ancestral population and is considered completed if the diverged populations remain reproductively isolated even after coming into secondary contact (Coyne & Orr, 2004; Dobzhansky, 1951; Mayr, 1942, 1963; Orr, 1996). However, many animal and plant species remain distinct entities in nature even if they occasionally hybridize and exchange genes in parapatry or sympatry (Barton & Bengtsson, 1986; cf. DeMarais et al., 1992; Gante et al., 2016; Mallet, 2007; Nolte & Tautz, 2010; Rieseberg et al., 2003; Trier et al., 2014). Research during the past decades has revealed that hybridization can have deleterious effects due to hybrid inferiority and negative epistasis in admixed genomes, but it may also be beneficial and fuel adaptive diversification and speciation by facilitating novel combinations of alleles that become targets of divergent selection (Arnold & Meyer, 2006; Berner & Salzburger, 2015; Fontaine et al., 2015; Saetre, 2013; Seehausen, 2004). As a consequence of recombination, and depending on the nature of selection and the genetic architecture of its targets, the effect of interspecific gene flow on the evolution and maintenance of reproductive isolation may vary even along individual genomes (Kim et al., 2018; Ravinet et al., 2017; Yeaman et al., 2016). Thus, the genomes of recently diverged species may represent mosaics consisting of genomic regions with significant differentiation (i.e., low or no effective gene flow) interspersed with less differentiated regions that experienced more effective gene flow (Nosil et al., 2009; Wu, 2001).

Sepsid flies (Diptera: Sepsidae) generally depend on decaying organic matter for reproduction and development, and have become a model for the study of sexual selection and ecological adaptation (Baur et al., 2020; Blanckenhorn, 1999; Blanckenhorn et al., 2000; Eberhard, 1999, 2002; Kraushaar & Blanckenhorn, 2002; Kraushaar et al., 2002; Parker, 1972a, 1972b; Pont & Meier, 2002; Puniamoorthy et al., 2009; Rohner et al., 2015; Rohner et al., 2016; Ward, 1983; Ward et al., 1992). While some species can show marked substrate specialisations, other species may regularly dwell on multiple resources ranging from vertebrate dung to rotting plant matter (Pont & Meier, 2002). In Europe several (up to 12) widespread species of the genus *Sepsis* with very similar ecological niches coexist in and around livestock faeces, often on the same pasture, an ubiquitous resource in many natural and managed agricultural grasslands worldwide (Rohner et al., 2015, 2019).

While the precise ecological niches of many species remain unclear (Blanckenhorn et al., 2020, 2021; Khelifa et al., 2019; Roy et al., 2018), the phylogeny of sepsid flies is well resolved (Su et al., 2008, 2016) and entails multiple pairs of closely related species that occupy similar niches. These species pairs provide excellent opportunities to explore the extent and genomic consequences of hybridization during speciation. One species pair of interest comprises *S. cynipsea* and *S. neocynipsea*, both of which exhibit a wide geographic distribution and occur in sympatry across major parts of their natural range in Europe. *Sepsis cynipsea* is the most abundant sepsid species in Central and Northern Europe and deposits its eggs mainly into fresh cattle dung. *Sepsis neocynipsea* is common throughout North America, where it occupies similar niches to those of *S. cynipsea* in Europe. While overall very rare in numbers but nevertheless widespread in Europe, *S. neocynipsea* can be locally common at higher altitudes, such as the Alps, where the species typically occurs in sympatry with *S. cynipsea* (Ozerov, 2005; Pont, 1987; Pont & Meier, 2002; pers. observation). Based on phylogenetic evidence and their worldwide distribution, it is yet unclear whether *S. neocynipsea* originated in North America and subsequently spread to Europe, where it appears to be marginalized to higher altitudes by the similar but competitively superior (and more common) *S. cynipsea*, or vice versa. Despite strong similarities in morphology and behaviour (Giesen et al., 2017), the two species (and the continental *S. neocynipsea* populations) are genetically distinct and show quantitative differences in mating system (Baur et al., 2020; Blanckenhorn et al., 2021; Pont & Meier, 2002; Rohner et al., 2016). Under laboratory conditions, heterospecific pairings resulted in lower copulation frequencies and longer copulation latencies than conspecific pairings, suggesting evidence of species recognition and pre-copulatory reproductive isolation. Females of both species discriminate more strongly against heterospecific males of sympatric vs. allopatric origin, and such assortative mating was found to be stronger in areas where the two species co-occur in sympatry in Switzerland (Giesen et al., 2017). However, our previous studies also indicate that *S. cynipsea* and *S. neocynipsea* can produce fertile hybrid offspring under laboratory conditions despite reduced fertility and fecundity of the F1 offspring. Species barriers thus appear to be mediated by assortative pre-copulatory mating behaviours, whereas there is little evidence for strong post-copulatory isolating barriers (Giesen et al., 2019). While these laboratory studies imply that interspecific gene flow can occur and varies with eco-geographic context in nature, we know little about actual levels of hybridization between these two species in nature. Here we therefore investigated the extent of gene flow between *S. cynipsea* and *S. neocynipsea* at natural sites where they now occur in sympatry, as compared to pairs of sites at which the species putatively occur in allopatry, by means of comparative population genomic analyses.

The objective of our study was to assess the extent and patterns of genome-wide admixture between *S. cynipsea* and *S. neocynipsea* in Europe by applying a version of the ABBA–BABA test for historical gene flow (Durand et al., 2011; Green et al., 2010; Soraggi et al., 2018) that can exploit genome-wide allele frequency data of single nucleotide polymorphisms (SNPs). To this end, we sequenced the pooled genomic DNA of *S. cynipsea* and *S. neocynipsea* males wild-caught at multiple sites across Europe. At some of these sites the species occur in sympatry (e.g., in the Swiss Alps or the French Cevennes); at other sites we only detected *S. cynipsea* alone (Pont & Meier, 2002). Based solely on the contemporary opportunity of admixture, we expected to find higher genome-wide levels of gene exchange between *S. cynipsea* and *S. neocynipsea* at sites of sympatry than between allopatric pairs of sites (e.g., Martin et al., 2014 or Nadeau et al., 2013, for *Heliconius* butterflies). By contrast, lower levels of inferred gene flow in sympatry than allopatry could either indicate that allopatric populations share a hitherto undisclosed evolutionary history involving post-split gene flow, or, conversely, that divergent selection has reduced effective gene flow at sites of sympatry (Butlin, 1995; Coyne & Orr, 2004; e.g. Giesen et al., 2017, 2019; Kulathinal & Singh, 2000; Massie & Makow, 2005; Noor, 1999). More generally, any documented geographic variation in interspecific contemporary or historical gene flow should reflect population variation in reproductive, environmental and/or selective conditions (cf. Blanckenhorn et al., 2020, 2021). To distinguish between the two scenarios of underappreciated gene flow between allopatric populations vs. a reduction of gene flow in sympatry due to selection, we first established the genealogical relationship among populations of both species in Europe by means of Approximate Bayesian Computation (ABC) and coalescent simulations, using an independent microsatellite dataset including many (but not all) of the same populations of both species (Baur et al., 2020). The best-supported population genealogies with gene flow provided hypotheses that we then tested using *D*-statistics applied to the pool-sequenced data. Our analyses provide the first detailed insight into the complex shared evolutionary history of *S. cynipsea* and *S. neocynipsea* in Europe.

**Table 1:** Description of sampled *Sepsis* populations

Species	ID	Location	Country	Type	Latitude	Longitude	Altitude (m)	Alignment with *S. thoracica* [%]	Average read depth
a) Wild-caught									
* S*. *cynipsea*	*PtC*	*Petroia*	*Italy*	Allo	43.23	12.56	570	48.1	29.2
	MoC	Le Mourier	France	Sym	44.06	3.43	900	49.5	62.5
	SoC	Sörenberg	Switzerland	Sym	46.81	8.06	1200	51.3	35.1
	ZuC	Zürich	Switzerland	Sym	47.40	8.57	450	51.3	37.3
	*PhC*	*Pehka*	*Estonia*	Allo	59.48	26.35	5	50.6	19.8
*S*. *neocynipsea*	MoN	Le Mourier	France	Sym	44.06	3.43	900	48.6	74.2
	GeN	Geschinen	Switzerland	Sym	46.50	8.27	1350	50.5	32.1
	HoN	Hospental	Switzerland	Sym	46.62	8.58	1500	52.3	25.4
	SoN	Sörenberg	Switzerland	Sym	46.81	8.06	1200	50.7	23.0
b) Laboratory iso-female lines									
*S*. *cynipsea*	IZuC	Zürich	Switzerland	Sym	47.40	8.57	402	50.1	19.2
*S*. *neocynipsea*	IZuN	Zürich	Switzerland	Sym	47.40	8.57	402	50.0	67.2
c) *S*. *orthocnemis*		Lenzerheide	Switzerland		46.73	9.56	1470	46.9	13

Table shows the “ID”, “Location”, and “Country” of (a) wild-caught *S*. *cynipsea* and *S*. *neocynipsea* populations plus (b) two samples of populations maintained in the laboratory. A third species, *S*. *orthocnemis* (c), was used as a reference outgroup in our analyses. Our dataset includes populations that occur either in sympatry or in allopatry with populations of the other focal species as shown in the column “Type”

## Materials and Methods

### Sample Collection and Fly Cultures

**Fig. 1:**
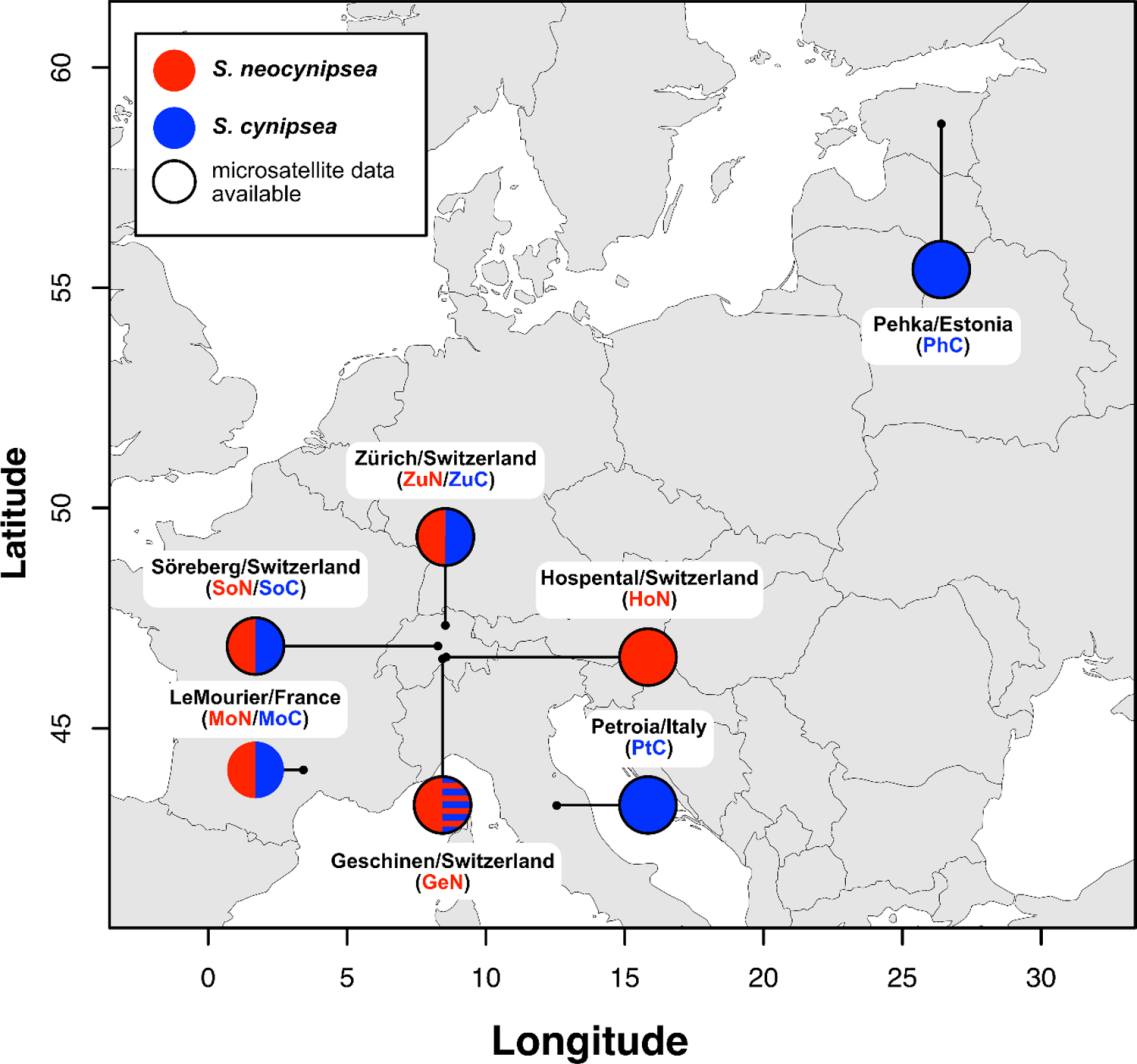
Sampling locations of *S. cynipsea *(blue) and *S. neoocynipsea* (red) populations in Europe used in this study (with acronyms). Sampling sites with independent complementary microsatellite data from a previous study (Baur et al., 2020) are highlighted by a black outline. For Geschinen (Switzerland), only microsatellite genetic data for both species were available, as highlighted by the dashed blue half circle (Color figure online)

We studied inter- and intraspecific gene flow using genomic data of sympatric *S. cynipsea* and *S. neocynipsea* populations from two higher-altitude sampling sites in the Swiss Alps (Sörenberg) and the French Cevennes (Le Mourier), and from one low-altitude site in Zürich (Fig. 1; Table 1). These pairs of sympatric populations were complemented with two geographically distant European populations of *S. cynipsea* from Umbria, Italy (Petroia) and Estonia (Pehka), where *S. neocynipsea* does not occur (Pont & Meier, 2002; Fig. 1; Table 1). We refer to these populations from Petroia and Pehka as allopatric. Both the literature (Pont & Meier, 2002) and our previous sampling experience suggest that *S. neocynipsea* is rare (or cannot be readily found) at low altitudes in Europe (except Zürich). To increase the number of *S. neocynipsea* samples, we obtained two further high-altitude *S. neocynipsea* populations in Switzerland where *S. cynipsea* also occurs (Geschinen and Hospental; Fig. 1; Table 1). In Hospental we also found *S. cynipsea* but did not catch sufficient individuals for pooled resequencing. For each wild-caught sample we randomly selected 50 males and jointly extracted DNA from the pooled sample for sequencing. We focused on males only because females of the two species cannot be distinguished based on morphological traits alone (Pont & Meier, 2002).

Altitude (as shown in Table 1) might co-vary with ecological factors affecting interspecific gene flow. In our sampling we therefore included two sympatric sites from higher altitudes (Le Mourier and Sörenberg) plus an additional low altitude sympatric site in Zürich (Fig. 1; Table 1). Contrary to those wild-caught specimens from higher altitudes, however, the Zürich flies stemmed from laboratory iso-female lines that had been initiated from a single wild-caught female two years prior to sequencing. We propagated the offspring of these females in the laboratory at census population sizes ranging from 10 to 100, and equally pooled the DNA of 50 males from each sample for whole-genome sequencing (Table 1; see Supplementary Text S1 for technical details on the laboratory populations). We further pooled 10 individuals from an inbred strain of the closely related *S. orthocnemis* collected from near Lenzerheide, Switzerland, and used these 10 individuals collectively as the phylogenetic outgroup in subsequent analyses (Table 1).

### DNA Library Preparation and Next Generation Sequencing

Pooled re-sequencing (Pool-Seq; Schlötterer et al., 2014) has proven powerful to reliably estimate allele frequencies of population samples (e.g., Kapun et al., 2020, 2021; Lynch et al. 2014; Zhu et al., 2012). We therefore pooled 50 individuals per sample prior to whole-genome DNA extraction using the UltraPure Phenol:Chloroform:Isoamyl alcohol (25:24:1, v/v) extraction kit (Thermo Fischer Scientific, Waltham, USA) according to the manufacturer’s protocol. Quantification of genomic DNA was performed with a Qubit Fluorometer (Thermo Fischer Scientific), and library preparation was carried out with the TruSeq DNA PCR-Free Library Preparation kit (Illumina, San Diego, USA) according to the manufacturer’s protocol. Fragment-size distributions of all libraries were validated on a TapeStation 2200 (Agilent Technologies, Waldbronn, Germany). Paired-end sequencing to 126 or 150 bp (MoC and MoN, only) read lengths was conducted on an Illumina HiSeq 2500 version 4 sequencer after labelling and pooling the barcoded DNA onto one lane to achieve a genome-wide read-depth of ca. 20–60 fold per pooled DNA library, which corresponds to an average read depth of 0.4–1.2 fold per pooled individual.

### Mapping Pipeline and SNP Calling

Qualitative validation of sequence data before and after trimming was performed with FastQC v. 0.11.4 (Andrews et al., 2011). After removal of Illumina-specific adapters and trimming with Trimmomatic v. 0.36 (Bolger, Lohse & Usadel, 2014), the *S. cynipsea, S. neocynipsea* and *S. orthocnemis* reads were mapped to the draft genome of *S. thoracica*, another closely related sepsid species, with bwa mem﻿ v. 0.7.12 (Li & Durbin, 2009). The *S. thoracica* sample used for genome sequencing was collected near Capriasca, Ticino, Switzerland. The draft genome v.0.1 was built from Oxford Nanopore long reads (ca. 25× read depth), assembled with Canu (Koren et al., 2017), and polished with Illumina short reads (ca. 20× read depth) with Pilon v.1.22 (Walker et al., 2014). The assembly used in this study is available under the rules of the Fort Lauderdale Agreement (https://www.sanger.ac.uk/wp-content/uploads/fortlauderdalereport.pdf) from http://www.cgae.de/seto_01_genome.fasta and http://www.cgae.de/seto_01_genome_masked.fasta. Despite using a PCR-free sequencing library preparation kit, we employed PCR duplicate removal with Picard v. 1.109 (http://broadinstitute.github.io/picard/), which is part of our standard mapping pipeline, and we realigned reads around indels in each raw alignment file using RealignerTargetCreator and IndelRealigner from GATK v. 3.4–46 (McKenna et al., 2010). Only reads with a PHRED-scaled mapping quality of 20 or more were retained. We compiled the aligned reads from all population samples into a single mpileup file that collects the allelic information of all genomic positions and samples using samtools﻿ v. 1.3.1 (Li et al., 2009).

The variant caller Pool-SNP (Kapun et al., 2020, 2021) was used to identify high-confidence SNPs with the following combination of heuristic SNP-calling parameters: coverage of each Pool-Seq sample ≥ 10×; coverage of each Pool-Seq sample ≤ 95% percentile of coverage distribution across contigs and samples; minimum allele count of a minor allele at a SNP across all combined Pool-Seq samples > 20×; minor allele frequency at a SNP across all combined Pool-Seq samples > 0.01. Only SNPs for which all samples fulfilled the above criteria were retained. SNPs located within repetitive regions and within 5 bp distance to indels that occurred in more than 20 copies across all pooled samples were removed to avoid paralogous SNPs due to mis-mapping around indel polymorphisms. The resulting VCF file was converted to the SYNC file format (Kofler et al., 2011), and a custom Python script was used to calculate sample-specific allele frequencies for major alleles at each SNP (sync2AF.py;https://github.com/capoony/DrosEU_pipeline).

### Demographic Inferences for *S. cynipsea* and *S. neocynipsea* in Europe

Tests for historical gene flow that rely on detecting patterns of derived allele sharing inconsistent with a supposed species or population genealogy require prior knowledge about this genealogy. To gain such prior knowledge and inform our tests for interspecific gene flow between *S. cynipsea* and *S. neocynipsea* (see below), we first inferred a baseline demographic history of the two species in Europe using Approximate Bayesian Computation (ABC; Beaumont, 2019) as implemented in DIYABC v.2.1.0 (Cornuet et al., 2014). For this purpose, we re-analysed an independent population-genetic dataset by Baur et al. (2020) comprising 14 European *S. cynipsea* and 6 European *S. neocycnipsea* populations that were genotyped at 9 microsatellite loci. These microsatellites only represent a tiny fraction of the genome and hence do not reflect fine-scale variation in gene flow along the genome. However, this microsatellite dataset avoids two shortcomings of our whole-genome pool-sequencing dataset, namely the lack of individual genotypes and the elevated sampling noise due to pooling DNA from multiple individuals before library preparation (e.g., Cutler & Jensen, 2010). Moreover, small to moderate numbers of microsatellites have repeatedly been shown to yield plausible demographic inferences, including estimates of migration rates (e.g., Aimé et al., 2014; Caracristi & Schlötterer, 2003; Kaya et al., 2021; Leblois et al., 2014; Schlötterer et al., 2006).

From the 20 populations in the microsatellite dataset of Baur et al. (2020; see BaurEtAL_2021_MicSat_Raw.xlsx in Supplementary Material) we chose those 7 populations that are also represented in our present whole-genome pool-sequencing dataset (Fig. 1). For *S. cynipsea* these are the populations from Pehka (Estonia; denoted as PhC), Petroia (Italy; PtC), Zürich (Switzerland; ZuC), and Sörenberg (Switzerland; SoC), and for *S. neocynipsea* the populations from Geschinen (Switzerland; GeN), Sörenberg (SoN), and Zürich (ZuN). For *S. cynipsea* we additionally included a fifth population from Geschinen (GeC). The GeC population is not represented in our whole-genome dataset, but its inclusion in the microsatellite dataset meant that our sampling design for the ABC demographic analyses was balanced in the sense that it comprised data from both species for all three Swiss sites (Geschinen, Sörenberg, Zürich). To establish a baseline genealogy for all these 8 European *S. cynipsea* and *S. neocynipsea* populations, we assumed a basal split of *S. cynispsea* and *S. neocynipsea* at time ${t}_{cn}$, followed by species-specific splits that gave rise to the respective 5 and 3 sampled populations of each species. As the joint number of possible topologies for the two subtending population trees is too large (945 × 3 = 2835) for an exhaustive ABC-type model comparison, we first defined an initial set of species-specific candidate population-tree topologies based on linearised pairwise ${F}_{ST}$ (Goudet et al. 2005) computed at all 9 microsatellites (Supplementary Text S2, Fig. S1). For *S. cynipsea*, pairwise ${F}_{ST}$ suggested that PhC and PtC diverged from the common line of descent earlier than any of the three Swiss *S. neopcynipsea* populations GeN, SoN, and ZuN (Fig. S2A). Individual candidate topologies however differ by whether PhC and PtC find a common ancestor before they merge with the common ancestor of the Swiss populations. We assigned to each branch in the topology an effective population size, and to each population a split time before present. We then compared the fit of the resulting species-specific demographic scenarios (Figs. S2A and S2B) to the data using ABC model comparison (Supplementary Text S3; Table S1). After identifying the best-fitting demographic models for each species, we combined these species-specific models into four joint demographic scenarios including both species (Fig. S2C; Table S2). For *S. cynipsea*, scenario 1 is the only one in which PhC and PtC do not find a common ancestor before they merge with the common ancestor of the three Swiss *S. neocynipsea* populations (GeN, SoN, ZuN). In contrast, scenarios 2 to 4 assume that PhC and PtC are more closely related to each other than to any of GeN, SoN, and ZuN. Scenarios 2 to 4 only differ in the relationships among GeN, SoN, and ZuN (Fig. S2C). We again compared the fit of these four scenarios to the data using ABC (Supplementary Text S3). For the best-fitting joint scenario we also estimated the effective population sizes and split times using ABC (Supplementary Text S3).

To complement the ABBA–BABA tests for admixture described below, we included discrete pulses of bidirectional interspecific gene flow to a pruned version of the population genealogies pertaining to the demographic scenario without gene flow that best fitted the microsatellite data from Baur et al. (2020). Pruning here means that we removed all populations but those included in our four-population tree used in the respective ABBA–BABA test as described below (i.e. populations P1, P2, P3, P4) to detect gene flow between either P1 and P3 or P2 and P3. Specifically, we modelled two concurrent pulses of bidirectional gene flow at ${t}_{a}$ generations into the past: one in which a proportion ${m}_{1\leftrightarrow 3}$ of individuals in P1 is replaced by immigrants from P3 and vice versa, and another one in which a proportion ${m}_{2\leftrightarrow 3}$ of individuals in P2 is replaced by immigrants from P3. We jointly estimated effective population sizes, population split times, the time of gene flow, and the admixture proportions using ABC (see Supplementary Text S3 for details; Fig. S3).

### Testing for Genome-Wide Signals of Interspecific Gene Flow

**Fig. 2:**
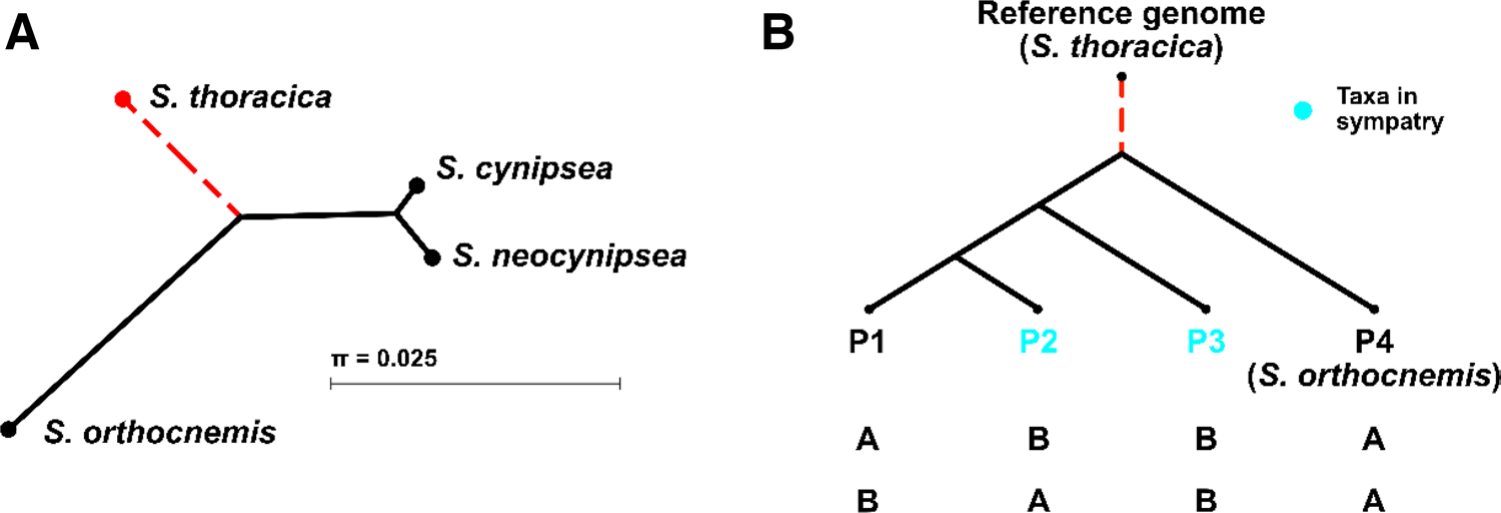
Putative phylogeny of sepsid species studied. **A** Species phylogeny inferred from alignments of nucleotide sequences at the cytochrome oxidase subunit II (CO-II) mitochondrial locus (Genbank sequences from Su et al., 2016) with the Neighbor-Joining method implemented in CLC Main Workbench (v. 8.1.2; https://www.qiagenbioinformatics.com/products/clc-main-workbench/). The tree topology is consistent with the phylogeny by Su et al.’s (2016) combining multiple nuclear and mitochondrial markers. *Sepsis orthocnemis* was used as the outgroup for the focal species *S. cynipsea* and *S. neocynipsea*. Whole-genome sequencing reads from all three species were aligned to the *S. thoracica* reference genome (dashed red branches). Branch lengths are proportional to the mean number of pairwise sequence differences *π*. **B** Generic species tree assumed in all our ABBA–BABA tests for gene flow among triplets of *S. cynipsea* and *S. neocynipsea* populations from various sites in Europe

To test for interspecific gene flow, we used the ABBA–BABA test for admixture in the presence of incomplete lineage sorting (Durand et al., 2011; Green et al., 2010). We provide a brief motivation of the approach in the following and refer to Supplementary Text S4 for details. Imagine a rooted phylogeny with four species (P1 to P4) and a topology of (((P1, P2), P3), P4) as illustrated in Fig. 2B. In an alignment of one haploid genome from each species, incomplete lineage sorting under neutral evolution leads to two mutational configurations of the ancestral (A) and derived (B) allele, ABBA and BABA, that are incompatible with the species topology (Fig. 2B) under the infinite-sites model of mutation (Kimura, 1969). In the absence of gene flow between P2 and P3, ABBA and BABA configurations occur with equal probability (Hudson, 1983; Tajima, 1983). In contrast, historical gene flow between P2 and P3 causes an excess of ABBA configurations (Durand et al., 2011; Green et al., 2010). This logic is captured by the *D*-statistic, a scaled difference across a set of bases between counts of ABBA and BABA configurations bounded by − 1 and + 1 (Green et al., 2010). The ABBA–BABA test examines significant deviations of *D* from 0. A significant excess of ABBA suggests evidence for gene flow between P2 and P3. A significant depletion of ABBA is equivalent to an excess of ABBA if the positions of P1 and P2 in the species topology are swapped, and hence either suggests gene flow between P1 and P3, or a reduction in gene flow between P2 and P3 relative to gene flow between P1 and P3.

The original version of the ABBA–BABA test and modifications of it have been applied to different types of genome-scale polymorphism data obtained with various sequencing strategies, including whole genome sequencing (e.g. Green et al., 2010), RAD sequencing (e.g., Eaton & Ree, 2013; Meier et al., 2017; Streicher et al., 2014), and exon capture data (e.g., Heliconius Genome Consortium, 2012). More recently, Durand et al. (2011) and Soraggi et al. (2018) extended the original test to allele frequency data, hence to unphased sequencing data (including Pool-Seq data; Schlötterer et al., 2014). We implemented the extensions by Durand et al. (2011) and Soraggi et al. (2018) in a Python script (https://github.com/nhmvienna/ABBABABA-4AF) and refer to the respective test statistics as *D*_D_ and *D*_S_. Our script also computes jackknifed *z*-scores based on a matrix of allele frequencies for previously defined high-confidence SNPs (see Supplementary Text S4 for details). We adopted the commonly used significance threshold of |*z*| > 3 (Reich et al., 2011; Jeong et al., 2016; Novikova et al., 2016) to identify significant deviations of *D*_S_ and *D*_D_ from zero.

We were concerned that using the same genome assembly both as the reference for read mapping and as the outgroup (P4) in the ABBA–BABA tests could lead to biased estimates of gene flow. We therefore mapped the three ingroup and the outgroup species against the reference genome of yet another species, *S. thoracica*. A phylogenetic analysis based on CO-II sequences by Su et al. (2008) indicated that *S. thoracica* is approximately equally related to *S. cynipsea* and *S. neocynipsea* as it is to the outgroup species *S. orthocnemis* (Fig. 2A). We therefore expected that reads of the ingroup and the outgroup species would map equally well to the reference species, which is indeed what we observed (Table 1).

We focused our analysis of interspecific sympatric sampling gene flow on the three sites Zürich, Sörenberg, and Le Mourier (Table 1). In all ABBA–BABA tests, we positioned the two focal populations as P2 and P3 ingroups in the phylogeny in both possible orders and used various P1 ingroup populations (Fig. 2B) assumed to occur in allopatry with the P2 populations. We used various P1 ingroups because Durand et al. (2011) showed that the choice of P1 can influence the test results. We then obtained window-wise estimates of gene-flow by calculating *D*_S_ and *D*_D_ in genomic windows of 500 consecutive SNPs (see also Supplementary Text S4) and computed genome-wide statistics by averaging across all windows.

## Results

### The Estimated Evolutionary History of *S. cynipsea* and *S. neocynipsea* in Europe

**Fig. 3:**
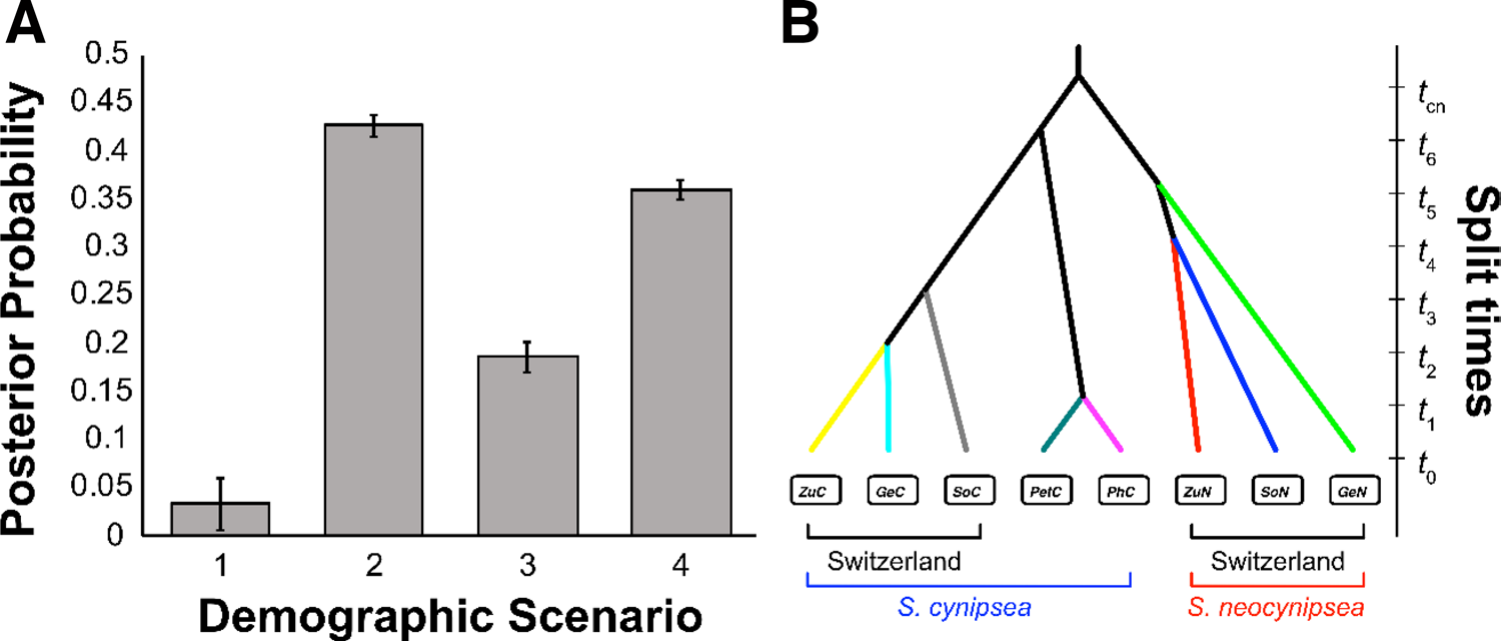
Inference of population genealogy and demography for two European sepsid sister species. **A** Posterior probabilities of 4 demographic scenarios inferred by Approximate Bayesian Computation (ABC) based on microsatellite data and one million coalescent simulations per scenario (Table S2). **B** Population genealogy of the best fitting demographic scenario 2 (Fig. S2). Time ${t}_{cn}$ refers to the splitting time of *S. cynipsea* and *S. neocynipsea*. The population split times ${t}_{i}$ on the y-axis are not drawn to scale. For parameter estimates see Table S3

Our analyses of population differentiation (by pairwise ${F}_{ST}$) across all allo- and sympatric populations of the two species based on microsatellite data from Baur et al. (2020) suggested that the three Swiss *S. cynipsea* populations from Geschinen (GeC), Sörenberg (SoC) and Zürich (ZuC) are genetically more similar to each other than any of them is to the populations from Pehka (PhC, Estonia) and Petroia (PtC, Italy; Fig. S1), as expected from geographic distances among populations (Fig. 1). Somewhat surprisingly, however, pairwise ${F}_{ST}$ further suggested that the Swiss higher-altitude populations of both species from Sörenberg (SoC & SoN) are genetically more similar to the conspecific Swiss low-altitude populations from Zürich (ZuC & ZuN) than to the geographically closer high-altitude populations from Geschinen (GeC & GeN; Fig. S1). Coalescent simulations and ABC inference conducted to more thoroughly assess the evolutionary relationships among these populations favoured scenario 2 in Fig. S2C (Fig. 3A; median posterior probability [MPP]: 0.42; 95% confidence interval [CI]: [0.41, 0.44]; Table S2). In this scenario, the two Swiss high-altitude *S. cynipsea* populations (GeC & SoC) find a common ancestor before merging with the low-altitude Zürich population (ZuC), whereas for *S. neocynipsea* the high-altitude Sörenberg population SoN still finds a common ancestor with the low-altitude Zürich population ZuN before this ancestral population merges with the second high-altitude population GeN (Fig. 3B), consistent with the pairwise ${F}_{ST}$ results in Fig. S1. Moreover, in scenario 2, the northern PhC and the southern PtC populations find a common ancestor before either of them merges with the basal *S. cynipsea* lineage that apparently gave rise to the Swiss populations (GeC, SoC, ZuC; Fig. 3B; Fig. S2). Together these results informed our subsequent ABBA–BABA tests for interspecific gene flow in two ways. First, for *S. cynipsea* the Swiss and the non-Swiss populations find a common, ‘local’ ancestral population before these two ancestral populations merge more deeply in the past. Second, for *S. neocynipsea* the more geographically remote ZuN and SoN find a common ancestor before any of them merges with GeN. In both species there is therefore no congruent association between phylogenetic and geographic distance.

Parameter estimates obtained with ABC under scenario 2 revealed similar effective population sizes (*N*_e_) for all Swiss populations of *S. cynipsea* (median posterior *N*_e_ ranging from 12,000 to 18,500) and *S. neocynipsea* (median posterior *N*_e_ ranging from 2600 to 18,700; Table S3). Only the most northern *S. cynipsea* population, PhC, exhibited a markedly lower *N*_e_ of 405 (95% equal-tail posterior credible interval [PCI]: [130 to 17,000]), which might be explained by this population being close to the northern margin of the species’ distribution. We note that posterior means for *N*_e_ of our eight populations were 3.6 to 6.5 times higher than their respective posterior medians, and both point estimates fell substantially closer to the respective lower bounds of the 95% PCI than to the upper bounds (Table S3). These observations are consistent with an observed asymmetry of the posterior distributions for these parameters.

We found splitting times among Swiss populations of *S. cynipsea* to be very recent, dating back only 19 (PCI: [10, 110]) to 37 (PCI: [13, 223]) generations (*t*_2_ and *t*_3_; Fig. 3B). By contrast, the Italian (PtC) and Estonian (PhC) *S. cynipsea* populations were estimated to have split ca. 127 generations ago (PCI: [100, 433]; *t*_1_ in Fig. 3B), and the ancestral lineages of the Swiss and non-Swiss populations ca. 419 generations ago (PCI: [75, 4290]; *t*_6_ in Fig. 3B). The Swiss *S. neocynipsea* populations split around 104 (PCI: [24, 562]) to 283 (PCI: [51, 917]) generations ago from their common ancestors (*t*_4_ and *t*_5_; Fig. 3B and Table S3). Moreover, the median split time of the two species (*t*_cn_) was inferred as merely 2100 generations ago (PCI: [396, 16,000]), which further emphasizes the close relatedness of these two sister species. However, as for *N*_e_, posterior means of the split times were higher (1.2 to 1.9 times) than the respective posterior medians, and means and medians were much closer to the lower than the upper 95%-PCI bounds (Table S3). Overall, our point estimates of the demographic parameters indicate very recent species and population divergence, although credible intervals suggest that these point estimates might considerably underestimate divergence times and effective population sizes.

### Signals of Reduced Gene Flow in Sympatric Swiss Alpine and Subalpine Regions

The ABBA–BABA test for gene flow aims to detect an asymmetry in two alternative patterns of derived allele sharing among ingroup populations that are both inconsistent with the putative topology of an asymmetric four-population genealogy (Fig. 2B). Based on the population genealogy inferred with ABC (Fig. 3B), we ran a series of ABBA–BABA tests to find evidence for differences in accumulated interspecific gene flow among sympatric vs. allopatric populations (Table 1). The majority of our ABBA–BABA tests for gene flow at the Sörenberg site revealed a genome-wide deficiency of derived-allele sharing (i.e., ABBA patterns) between the local *S. cynipsea* and *S. neocynipsea* populations (Table 2A), suggesting that interspecific gene flow in sympatry (between P2 and P3) was overall lower than interspecific gene flow among putatively allopatric populations (P1 and P3; cf. Fig. 2B). This observation is robust to our choice of the P1 ingroup population, but sensitive to the version of *D*-statistic used (*D*_S_ vs. *D*_D_). We first conducted tests involving two (sympatric) *S. neocynipsea* populations from the Swiss Alps (GeN and HoN; Table 1) as putatively allopatric P1, with the sympatric species pair from Sörenberg as P2 (SoN; *S. neocynipsea*) and P3 (SoS; *S. cynipsea*). We observed a significant deficiency of ABBA patterns in all configurations (*D*_S_ > 0; Table 2A1, upper part), suggesting overall evidence for more gene flow among putatively allopatric than sympatric interspecific population pairs. While GeN and HoN are both separated by high mountains (altitudes ranging from ca. 600–3200 m) from the Sörenberg site, they are still geographically close (ca. 67.5 km and ca. 46.7 km Euclidean distance, respectively) and occur in sympatry with other sepsid species including *S. cynipsea*. We were therefore concerned that our assumption of no gene flow between P1 and P3 was violated, because GeN and HoN are parapatric rather than truly allopatric to our focal Sörenberg populations.

To address the concern that GeN and HoN might not be allopatric to SoC and SoN, we conducted additional ABBA–BABA tests involving presumably truly allopatric P1 populations from geographically distant sites in Europe. In this second series of tests we assigned the two remote *S. cynipsea* populations PhC and PtC as allopatric P1 ingroups (Figs. 1 and 2B; Table 1). Contrary to our expectation based on the much greater geographical distance between P1 and P3 (ca. 1850 km and ca. 550 km for PhC and PtC, respectively) than between P2 and P3 in these configurations, we still found a significant deficiency of ABBA patterns in both tests with *D*_S_ (*D*_S_ > 0; Table 2A1, lower part). In agreement with our previous tests involving GeN or HoN as P1, these additional tests thus equally suggest reduced amounts of accumulated interspecific gene flow among sympatric (P2 and P3) than among allopatric populations (P1 and P3). As we are much more confident that PhC and PtC represent truly allopatric P1 ingroups relative to P2 and P3, the deficiency of ABBA patterns at the Sörenberg site suggests a potentially recent local reduction in interspecific gene flow in sympatry.

**Fig. 4:**
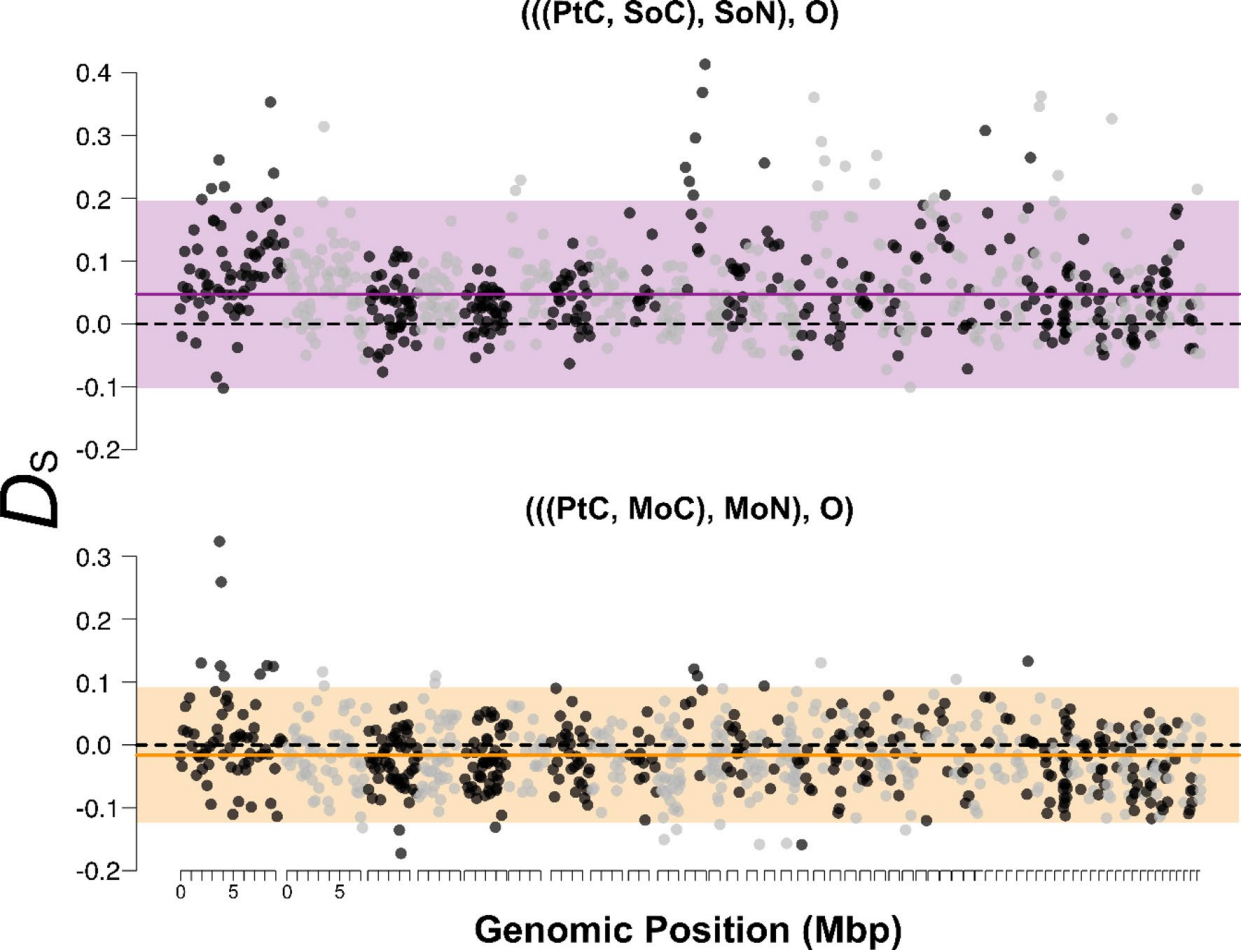
Variation of *D*_s_ along the genome. Dots indicate *D*_S_ (Soraggi et al., 2018) for genomic windows of 500 consecutive SNPs, oriented by contigs of the *S. thoracica* reference genome with a length of at least 50,000 bp (highlighted by alternating black and grey colors). The top and bottom panels show results for the population topologies used to test for interspecific gene flow at the focal sites Sörenberg (SoC–SoN; deficiency of ABBA patterns, in purple) and Le Mourier (MoC–MoN; excess of ABBA patterns, in orange). The horizontal dashed black and solid coloured lines indicate the neutral expectation (*D*_S_ = 0) and the mean genome-wide jackknife estimate, respectively. Coloured shading delimits mean *D*_S_ ± 2 standard deviations (based on windows of 500 SNPs) (Color figure online)

When repeating the ABBA–BABA tests with the *D*-statistic *D*_D_ suggested by Durand et al. (2011) with PhC and PtC as P1, we equally observed a significant deficiency of ABBA patterns, as with the *D*_S_ statistic (*D*_D_ < 0; *z*-scores − 8.4 and − 7.7, respectively; Table 2A2). With GeN or HoN as P1, the *D*_S_ statistic however indicated a non-significant slight excess of ABBA patterns (*D*_D_ > 0; *z*-scores 1.4 and 1.9, respectively; Table 2A2). Overall, all significant tests focussing on the Sörenberg site thus suggest a depletion of accumulated historical interspecific gene flow between the currently sympatric populations of the two sister species (SoC, SoN) relative to allopatric interspecific population pairs. We further observed that windows of elevated *D*_S_ were not homogeneously distributed along the genome, but rather aggregated in clusters in several contigs (Fig. 4, top).

Since both species commonly co-occur in alpine and some sub-alpine regions of Switzerland (Rohner et al., 2015), we were curious to see if a signal of relatively lower gene flow in sympatry in alpine regions was also evident at lowland sites of sympatry in this geographic area. We therefore additionally investigated a lowland site in Zürich where *S. cynipsea* and *S. neocynipsea* co-occur in sympatry. In contrast to the sequencing data from Sörenberg, which were generated from wild-caught specimens, the sequencing data from the Zürich site derive from a combination of natural and laboratory populations. Specifically, for *S. cynipsea* we sequenced a pool of flies sampled directly from the natural population as well as a pool of flies from a laboratory isofemale line derived from the same natural population earlier (see Material & Methods and Supplementary Text S1 for details), whereas for *S. neocynipsea* we only used a pool of flies from an isofemale laboratory line (Table 1). We found that ABBA–BABA tests were qualitatively robust to whether we used natural or laboratory populations. However, results again somewhat depended on the choice of the *D* statistic (*D*_S_ vs. *D*_D_) and the P1 ingroup (Table 2B). All ABBA–BABA tests based on *D*_S_ that included GeN, HoN, or PtC as P1 again revealed a significant deficiency of ABBA patterns, i.e. lower amounts of accumulated gene exchange among currently sympatric than allopatric populations. However, tests with the distant Estonian population PhC as P1 were not significant (Table 2B1). All tests based on *D*_S_ and involving sympatric pairs of populations from Zürich were therefore consistent with corresponding results for the Sörenberg site, consistently suggesting reduced interspecific gene flow in sympatry relative to allopatry. In contrast, only one out of four ABBA–BABA tests based on *D*_D_ was significant (Table 2B2), suggesting an excess of ABBA patterns. Overall, the majority of our significant results for the Zürich site support those for the Sörenberg site in suggesting potentially reduced levels of interspecific gene flow in sympatry.

Complementary to the ABBA−BABA analyses, we conducted ABC and coalescent simulations with and without gene-flow in sympatry and allopatry for the two Swiss sites Sörenberg and Zürich following the best fitting scenario 2. Consistent with the ABBA−BABA results, these analyses identified scenario 2B with reduced local gene flow (Table S4 & Fig. S3) as best-fitting for Sörenberg (MPP: 0.74, PCI: [0.73,0.75]) and Zürich (MPP: 0.59, PCI: [0.57,0.61]) when using PhC as the P1 ingroup. When using PtC as the P1 ingroup, scenario 2B was still best fitting for Sörenberg (MPP: 0.86, PCI: [0.85,0.87]; Table S4), whereas for Zürich scenario 2C without gene flow fitted best (MPP: 0.48, PCI: [0.47,0.49]; Table S4). However, the posterior probability of scenario 2B was only slightly lower (MPP: 0.34, PCI: [0.33,0.35]; Table S4). We further found that the estimated time *t*_a_ in generations since the migration event was low for the Sörenberg populations (median *t*_a_: 50, PCI: [11,361] and 88, PCI: [14,463] for analyses with PtC and PhC as P1 ingroups, respectively; Table S4), but higher for the Zürich populations (median *t*_a_: 416, PCI: [36, 898]; Table S4). In summary, the majority of our ABC inference analyses with gene flow supported our findings based on the ABBA–BABA tests in suggesting lower levels of interspecific gene flow in sympatry than allopatry. The ABC analyses further suggested that the sympatric interspecific pair of Sörenberg populations split about eight times more recently than the sympatric interspecific pair of Zürich populations .

### Signals of Elevated Gene Flow in Mid-altitude Sympatric Populations from Southern France

Analogous to the sympatric Swiss locations Sörenberg and Zürich, we further investigated a pair of sympatric *S. cynipsea* and *S. neocynipsea* populations from Le Mourier in the southern French Cevennes mountains (790 m), ca. 450 km southwest of the Sörenberg and Zürich sampling sites in Switzerland. Surprisingly, we found opposite patterns to those observed at the Swiss sites. All tests based on *D*_S_ showed a highly significant excess of ABBA patterns, hence *more* interspecific gene flow in sympatry than allopatry (Table 2C1). The corresponding tests based on *D*_D_ also indicated an excess of ABBA patterns, albeit not significant (Table 2C2). In contrast to the patterns for Sörenberg (Table 2A), we did not observe genomic clustering of extreme *D*_S_ values (Fig. 4, bottom).

**Table 2:** Results of ABBA–BABA tests based on *D*_S_ (top rows 1) or *D*_D_ (bottom rows 2) applied to *S. cynipsea* and *S. neocynipsea* pool samples from sympatric sites in (A) Sörenberg, (B) Zürich (isofemale line lab population), and (C) Le Mourier as P2 and P3 (or vice versa)

(A1)	P1	P2	P3	*D*_S_	SD (*D*_S_)	*z* (*D*_S_)
	GeN	SoN	SoC	0.008	1.35E − 03	5.8
	HoN	SoN	SoC	0.006	1.34E − 03	4.2
	**PhC**	**SoC**	**SoN**	**0.026**	**1.92E − 03**	**13.4**
	**PtC**	**SoC**	**SoN**	**0.039**	**1.97E − 03**	**19.8**

(A2)	P1	P2	P3	*−D*_D_	SD (*D*_D_)	*− z *(*D*_D_)
	GeN	SoN	SoC	− 0.007	5.09E − 03	− 1.4
	HoN	SoN	SoC	− 0.01	5.27E − 03	− 1.9
	**PhC**	**SoC**	**SoN**	**0.052**	**6.19E − 03**	**8.4**
	**PtC**	**SoC**	**SoN**	**0.049**	**6.34E − 03**	**7.7**

(B1)	P1	P2	P3	*D*_S_	SD (*D*_S_)	*z* (*D*_S_)
	**GeN**	**IZuN**	**IZuC**	**0.016**	**2.63E − 03**	**6.1**
	**HoN**	**IZuN**	**IZuC**	**0.017**	**2.68E − 03**	**6.5**
	PhC	IZuC	IZuN	0.001	3.89E − 03	0.3
	**PtC**	**IZuC**	**IZuN**	**0.012**	**3.74E − 03**	**3.3**

(B2)	P1	P2	P3	*− D*_D_	SD (*D*_D_)	*− z *(*D*_D_)
	**GeN**	**IZuN**	**IZuC**	**− 0.03**	**9.35E − 03**	**− 3.2**
	HoN	IZuN	IZuC	− 0.024	9.41E − 03	− 2.5
	PhC	IZuC	IZuN	0.014	9.77E − 03	1.4
	PtC	IZuC	IZuN	0.012	9.91E − 03	1.2

(C1)	P1	P2	P3	*D*_S_	SD (*D*_S_)	*z* (*D*_S_)
	**GeN**	**MoN**	**MoC**	**− 0.014**	**1.30E–03**	**− 11.0**
	**HoN**	**MoN**	**MoC**	**− 0.014**	**1.36E–03**	**−10.6**
	**PhC**	**MoC**	**MoN**	**− 0.030**	**1.84E–03**	**− 16.2**
	**PtC**	**MoC**	**MoN**	**− 0.016**	**1.93E–03**	**− 8.3**

(C2)	P1	P2	P3	*−D*_D_	SD (*D*_D_)	*z* (*D*_D_)
	GeN	MoN	MoC	0.011	5.03E − 03	2.3
	HoN	MoN	MoC	0.012	5.14E − 03	2.3
	PhC	MoC	MoN	0.010	5.37E − 03	1.9
	PtC	MoC	MoN	0.009	5.24E − 03	1.7

The upper halves of each group of 4 rows present tests using two sympatric *S. neocynipsea* populations from Geschinen (GeN) and Hospental (HoN) as P1 ingroups, while the lower halves present tests using two allopatric﻿ marginal European *S. cynipsea* populations from Pehka, Estonia (PhC) and Petroia, Italy (PtC) as P1 ingroups. The first three columns denote the phylogeny assumed for the test, with P4 always being *S. orthocnemis* (cf. Table 1C); the last three columns present genome-wide means of *D*_S_ and *D*_D_, their jack-knifed standard deviations SD(*D*_S_) and SD(*D*_D_), and the corresponding *z*-scores. Significant tests (z > 3) are bold. Given that *D*_S_ and *D*_D_ differ in sign, we report – *D*_D_ and – z(*D*_D_) for clearer comparison

## Discussion

Contrasting levels of interspecific gene exchange in contemporary sympatric vs. allopatric species pairs can reveal variation in the extent and nature of reproductive isolation, and thus provide insight into the underlying mechanisms of speciation. Our study explored this potential with genome-scale sequencing data from the two closely related dung fly sister species *S. cynipsea* and *S. neocynipsea.* The history of speciation in this system remains largely unknown despite detailed information on their ecology, morphology, life history, and behaviour (Blanckenhorn, 1999; Blanckenhorn et al., 2000, 2020, 2021; Eberhard, 1999, 2002; Giesen et al., 2017, 2019; Ozerov, 2005; Parker, 1972a,1972b; Pont & Meier, 2002; Puniamoorthy et al., 2009; Rohner et al., 2016; Ward, 1983; Ward et al., 1992).

Previous population genetic analyses of microsatellite markers (Baur et al., 2020) and hybridization experiments in the laboratory (Giesen et al., 2017, 2019) suggested that *S. cynipsea* and *S. neocynipsea* are genetically distinct but may occasionally hybridize in nature in sympatry. We here applied the ABBA–BABA test for historical gene flow to find genomic evidence for contemporary interspecific gene flow in sympatry and to relate it to levels of potential ancient gene flow between presently allopatric populations (Green et al., 2010; Durand et al., 2011; Soraggi et al., 2018). We analyzed patterns of DNA sequence variation among pools of field-caught and laboratory specimens from various sites (populations) across Europe at which either both species now occur in sympatry or only the more common and widespread species (*S. cynipsea*) occurs. As discussed in detail below, depending on the focal sympatric site, we found two qualitatively opposite patterns of interspecific gene exchange in sympatry versus allopatry: a relative reduction in genetic admixture in sympatry at two sampling sites in Switzerland, but also a relative excess of admixture in sympatry at a sampling site in Southern France.

Previous studies comparing levels of interspecific gene flow in sympatry and allopatry in other taxa found the full spectrum of results that we obtained for just a single species pair. Martin et al. (2014; see also Nadeau et al., 2013) and Brandvain et al. (2014; see also Grossenbacher & Whittall, 2011) found evidence for higher levels of interspecific baseline gene flow in sympatry than allopatry when studying *Heliconius* butterflies and monkey flowers (*Mimulus guttatus/nasutus*), respectively. In contrast, less gene flow in sympatry than allopatry was observed in studies of *Drosophila arizonae*/*mojavensis* (Massie & Makow, 2005) and two species of sea squirt (Tunicata: Bouchemousse et al., 2016), while no geographic variation in levels of gene flow between sympatric and allopatric populations was found in studies of wild tomatoes (Nakazato et al., 2010).

### Evidence that Low Gene Flow in Sympatry is Unlikely an Artefact of Ancient Population Structure

A reduction in average gene flow in sympatry compared to allopatry may suggest contemporary natural or sexual selection (Andersson, 1994) against hybridization that may ultimately lead to reinforcement and speciation (Butlin, 1995; Coyne & Orr, 2004; Noor, 1999), as indicated by premating behavioural barriers and fecundity or fertility reductions previously described for interspecific pairings between our two focal species by Giesen et al. (2017, 2019). Our evidence for differential interspecific gene flow between *S. cynipsea* and *S. neocynipsea* in sympatry vs. allopatry here relies on the ABBA–BABA test for admixture. This test requires defining a putative four-taxon genealogy and assumes that the ancestral groups giving rise to the admixing taxa were panmictic (cf. Fig. 2B). However, ancestral population structure can cause false positive signals of more recent, even contemporary admixture (e.g., Green et al., 2010, Theunert & Slatkin, 2017). While our study indicates reduced interspecific gene flow in sympatric populations of Swiss *S. cynipsea* and *S. neocynipsea*, similar genomic patterns may arise from differences in relatedness among populations of these two sister species (Theunert & Slatkin, 2017). To exclude confounding effects of their yet unknown population and species history, we supported our ABBA–BABA tests with ABC-based demographic inference from an independent microsatellite dataset of (largely) the same populations (Baur et al., 2020). These additional analyses provided insights into the evolutionary history of the two sister-species. First, we estimated the population sizes of both species to be of similar magnitude, suggesting that *S. neocynipsea* is probably more common in Europe than previously thought (Pont & Meier, 2002; Rohner et al., 2015, 2019). Second, *S. cynipsea* and *S. neocynipsea* likely split very recently, which is consistent with earlier reports of a close relationship between these species (Pont & Meier, 2002; Su et al., 2008, 2016). Third, the two presumably marginal northern and southern European *S. cynipsea* populations from Estonia and Italy find a common ancestor before their common ancestor merges with any of the three Swiss *S. cynipsea* populations. Moreover, all *S. cynipsea* populations find a common ancestor prior to merging with the three Swiss *S. neocynipsea* populations, as expected. Together, these findings suggest that the differences in interspecific gene flow identified by our ABBA–BABA tests are unlikely to be an artefact of a misidentified baseline evolutionary history of the two focal species in Europe.

Even though our ABC analyses comparing models of sympatric populations with and without migration qualitatively supported our ABBA–BABA test results, we caution that the coalescent simulator DIYABC merely allows modelling discrete migration pulses at a given time point, but not continuous levels of gene flow. We therefore do not assign too much weight to our estimates of the timing of gene flow. Unfortunately, this uncertainty about the temporal extent of gene flow also limits our resolution to categorise interspecific gene flow as occurring in sympatry vs. allopatry. For instance, sympatric populations that came into secondary contact very recently may have been allopatric before. A signal of reduced interspecific gene flow in sympatry may then simply reflect that the respective populations had not enough time yet to exchange genes. Conversely, somewhat more ancient colonization of a new location by at least one of the two species may have permitted interspecific gene flow at sites of sympatry, but there may not yet have been enough time for a reduction in gene flow to evolve (e.g. as a consequence of selection against hybridisation). Such a scenario could in principle explain the contrasting patterns of interspecific gene flow we observed at our sites of contemporary sympatry in the French Cevennes and Switzerland. Future demographic analyses of *S. cynipsea* and *S. neocynipsea* must therefore aim at resolving the spatio-temporal context of intra- vs. interspecific gene exchange of these two sepsid species in Europe and North America (Baur et al., 2020; Rohner et al., 2016).

### Variation in Sympatric Gene Flow Might be Linked to Climate, Species Abundance or Divergence in Reproductive Timing

Similar to research in *Heliconius* butterflies (Martin et al., 2014), we found evidence for an excess of average interspecific gene flow levels in sympatry compared to allopatry in our pool-sequenced sepsid field samples from Le Mourier, France (Table 1C). By contrast, at two Swiss sites, where the two species also occur in sympatry, we observed reduced average levels of such gene flow relative to interspecific pairs of contemporary allopatric populations (Table 1A, B), similar to patterns found for *Drosophila arizonae*/*mojavensis* (Massie & Makow, 2005) and sea squirts (Bouchemousse et al., 2016). We previously found laboratory evidence suggesting behavioural reinforcement when enforcing hybridization of *S. cynipsea* and *S. neocynipsea* (Giesen et al., 2017). This may indicate that selection preventing interspecific gene flow (Noor, 1999) might also occur in nature at least in our two sympatric Swiss populations. However, as evinced by our contrasting findings for the mid-altitude site in Le Mourier, reduced contemporary gene exchange is not a necessary outcome whenever *S. cynipsea* and *S. neocynipsea* coexist in nature, and does not appear to be linked to higher elevations. We thus speculate that the extent of contemporary (as opposed to historical) gene flow between these two dung fly sister species in sympatry varies with local environmental or selective conditions.

**Fig. 5:**
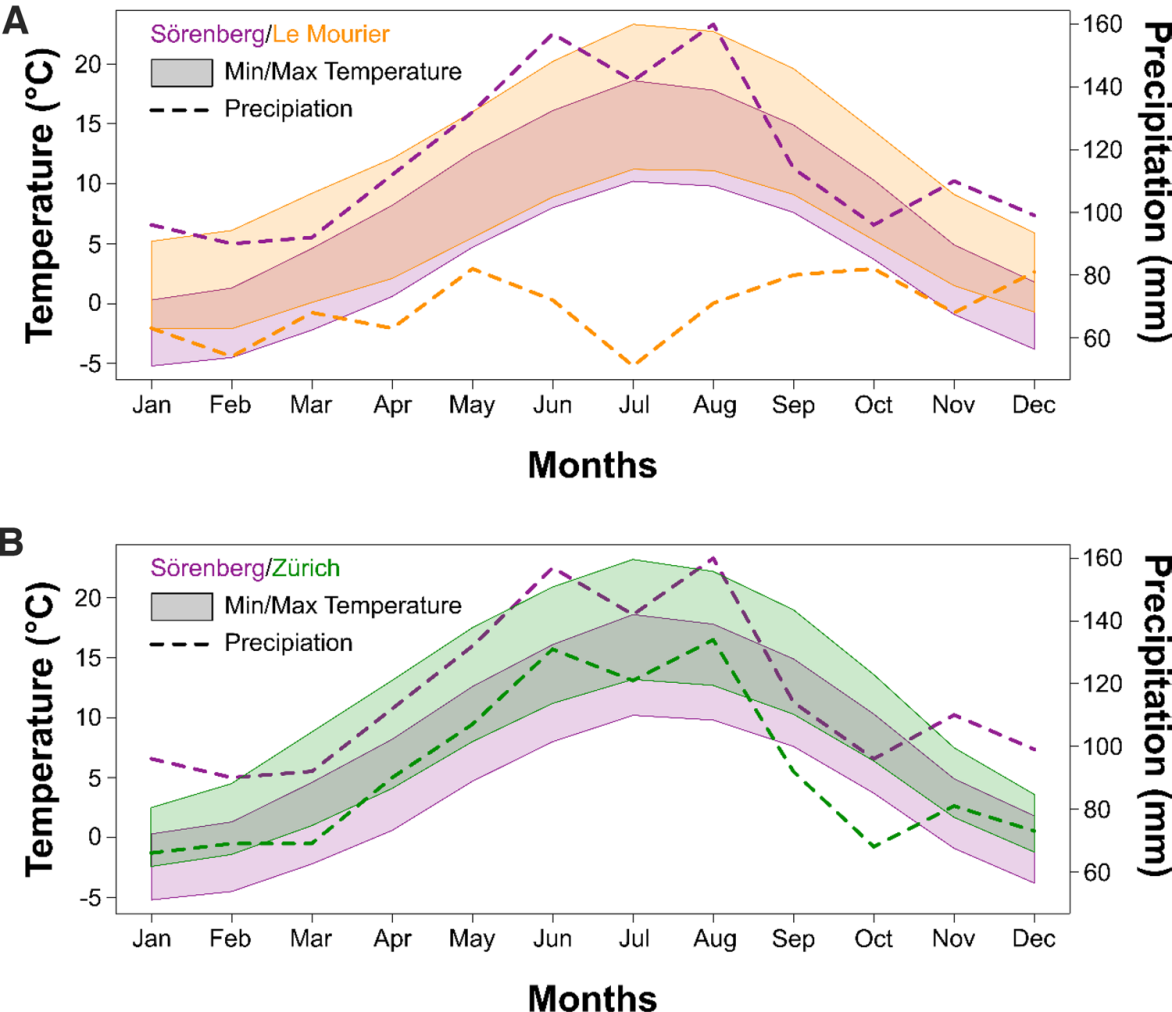
Climatic conditions at the sampling sites. Temperature (coloured polygons) and precipitation (dashed lines) differences between the mountainous Sörenberg (Swiss Alps; purple) and Le Mourier (French Cevennes; orange) sites (top), and between the alpine (purple) and subalpine (green) Swiss sites (bottom). These data from the WorldClim dataset (Hijmans et al., 2005) represent 50 year averages of observations in quadratic grid cells with an edge length of 2.5’ (i.e., ~ 5 km^2^ in area) around the coordinates of the two sites (Table 1) (Color figure online)

Well-documented differences in climate between Southern France and the Swiss Alps, and also between alpine and sub-alpine Swiss sites, could provide ecological explanations for the qualitative and quantitative differences in interspecific gene flow observed at the various locations (Doebeli & Dieckmann, 2003; Rohner et al., 2015; Fig. 5). While average minimum temperatures are similar in the French Cevennes and the Swiss Alps, average maximum temperatures are up to 5 °C higher at the French site compared to Sörenberg (Fig. 5). Additionally, these two sampling sites are characterized by very different precipitation regimes: during the main growing season from May until October, Sörenberg (a peat bog site) receives on average twice as much rainfall as Le Mourier. However, the precise mechanisms by which climate might affect intra- and inter-specific mating behaviour and/or success require further study in this system (Blanckenhorn et al., 2021; Giesen et al., 2017, 2019). We are also aware that our data are not replicated regarding any of the climatic variables mentioned, hence statistical power is limited. Thus, both more hybridization experiments under simulated environmental conditions in the lab and more sympatric vs. allopatric population pairs in Europe are needed to confirm our results and further elucidate the causes for the variation in sympatric gene flow found.

Other than driven by climate, differences in interspecific gene flow between the two Swiss sites might relate to species abundance and dispersal. *S. neocynipsea* is rare around Zürich, in fact in European lowlands north of the Alps in general (e.g. Pont, 1987), while it is more common at higher altitudes in the Alps (Pont & Meier, 2002; Rohner et al., 2015, 2019). We have no field data on potentially sex-biased dispersal in any sepsid species, which is difficult to study in such small insects, and found no evidence for either sex-specific differences in mobility under laboratory conditions (Mühlhäuser & Blanckenhorn, 2002; Teuschl et al., 2010) or strongly biased sex ratios across different seasons in the field in Switzerland (Rohner et al., 2019). Behavioural mechanisms preventing interspecific mating are expected to be stronger, and hence its evolution more likely, wherever frequencies of interspecific encounters are higher. Moreover, as our data for the Zürich site are based on both wild-caught and laboratory specimens, the weaker observed extent of gene exchange observed in Zürich relative to Sörenberg and Le Mourier might be partially explained by purifying selection in the laboratory against introgressed variants. However, our results from analyses of wild-caught and laboratory *S. cynipsea* samples from Zürich were consistent (Table 2B), suggesting that whether fly samples stem from nature or the laboratory does not strongly affect the outcome. Overall, we therefore do not think that purifying selection in the laboratory confounded our results.

Laboratory hybridization experiments with the same Swiss populations used here have previously shown that interspecific mating between *S. cynipsea* and *S. neocynipsea* occurs and can result in viable and fertile F_1_ hybrid females as well as offspring from backcrosses with the parental species (Giesen et al., 2017, 2019). Therefore phylogenetic, morphological and behavioural similarities of the two species may facilitate contemporary hybridization in nature in areas of co-occurrence, such as in the French Cevennes. In other areas, such as our sampling sites in Switzerland, subtle premating behavioural barriers and/or reductions in fecundity or fertility (Giesen et al., 2017, 2019) may combine with micro-ecological niche differences to mediate spatio-temporal divergence in reproductive timing, thus effectively preventing hybridization in nature. This interpretation is strengthened by a study on Central American *Archisepsis diversiformis* flies showing that mating between two disjunct populations from Panama and Costa Rica only occurred under forced laboratory conditions, while under conditions of free mate choice flies from the different populations did not interbreed (Eberhard, 2002; Puniamoorthy, 2014). Behavioural mating barriers therefore seem to evolve comparatively fast (Gleason & Ritchie, 1998; Puniamoorthy et al., 2009; Puniamoorthy, 2014), especially if species occur in sympatry and reinforcement by natural or sexual selection can operate (Coyne & Orr, 2004;  Garner et al., 2018; Ritchie, 2007; Seehausen, 2004).

We are not aware of many studies reporting empirical evidence for geographic variation in the extent of intraspecific gene flow in sympatry between two taxa in secondary contact. However, Riemsdijk et al. (2023) recently suggested a weaker species barrier between the toad species *Bufo bufo* and *B. spinosus* along a northern transect across the hybrid zone in France than along a southern transect. The authors hypothesised that this difference might be explained by a longer period of secondary contact in southern France. Yang et al. (2020) found lower, and more asymmetric, gene flow between two diverged lineages of wall lizards (*Podarcis muralis*) in the southernmost part of a contact zone in northern Italy compared to the northern parts of the zone. The authors showed that a suite of sexual traits that evolved recently in the southern part of the zone and that affects male-male competition is likely causing this difference. Meleshko et al. (2018) found that variation in interspecific gene flow in peatmosses (genus *Sphagnum*) was partially explained by variation in habitat quality (pH and mineral content). Even though we do not currently know what factors drive geographic variation in secondary-contact gene flow in our system, these examples suggest multiple potential reasons for variation in sympatric gene exchange between *S. cynipsea* and *S. neocynipsea*. Future research in this system should focus on identifying the causal factors.

### Extension of ABBA–BABA Tests Based on Allele Frequency Data to Pooled Re-sequencing

Our study of introgression patterns between sympatric *S. cynipsea* and *S. neocynipsea* populations relied on the ABBA–BABA test, which was initially designed for the analysis of haploid genotypes of one individual from each of four lineages (Green et al., 2010; Fig. 2B). Recent extensions of this original approach to allele frequency data by Durand et al. (2011) and Soraggi et al. (2018) enabled us to apply the method analogously to pool-sequenced laboratory and field-caught specimens (see also Deitz et al., 2016). As a technical innovation, we here provide a Python implementation of the ABBA–BABA approaches by Durand et al. (2011) and Soraggi et al. (2018) that takes a simple 2D matrix of allele frequencies as input (available at https://github.com/nhmvienna/ABBABABA-4AF; see also Supplementary Material). This implementation allows restricting the ABBA–BABA test to high-confidence SNPs that pass a set of user-defined filters. Such filtering can reduce artefacts of pool-sequencing (Schlötterer et al., 2014), which may produce false polymorphisms due to sequencing errors (Cutler & Jensen, 2010; Futschik & Schlötterer, 2010). In addition, and in contrast to the software package ANGSD (Korneliussen et al., 2014), our implementation accommodates an outgroup (P4) taxon different from the one used as a reference to call the SNPs. This feature reduces potential reference bias when the ingroup taxa (P1, P2, P3) strongly differ from an outgroup that at the same time serves as the reference (Ballouz et al., 2019).

While a formal comparison of the two existing ABBA–BABA statistics *D*_S_ and *D*_D_ was outside the scope of our study, we observed differences in the performance between them. We found that *D*_D_ of Durand et al. (2011) less often yielded significant deviations from zero than the *D*_S_ statistic of Soraggi et al. (2018). In our analyses of data from laboratory isofemale lines from the Zürich site we even obtained contradictory results for *D*_D_ and *D*_S_. However, in this case the corresponding *z*-score (z = 3.2) was very close to the significance threshold of |z| = 3. We also found that variances of *D*_D_ estimated by jackknifing were up to one order of magnitude larger than those of *D*_S_ (Table 1; Fig. S4). This behaviour indicates differences in the robustness of the two statistics, but ultimately is no sufficient basis for deciding which statistic to use or trust more (see also Supplementary Text S4). We therefore offered results based on both statistics.

In conclusion, we found evidence that the extent of interspecific gene flow between two closely related sepsid fly species in contemporary sympatry relative to allopatry varies with geographic location. Interspecific gene flow in sympatry appears to be reduced at two Swiss sites, where the two species might have locally adapted to different (micro-)ecological niches, such as different breeding times or phenologies, and/or where some pre- or postmating reproductive barriers might have evolved (Giesen et al., 2017, 2019). However, most European sepsid species are rather widespread habitat generalists with similarly broad thermal performance ranges (see e.g. Khelifa et al., 2019), so we deem their potential for local adaptation limited. Future studies are needed to determine if ecology, climate, and/or sexual selection are indeed driving the admixture patterns we identified, and whether the observed gene flow is contemporary or ancient. Future research should analyse patterns of genomic differentiation across more sympatric and allopatric sepsid populations from Europe and North America (Baur et al., 2020). It still remains unclear why *S. neocynipsea* is rare in Europe but common in North America (Rohner et al., 2015, 2016; Baur et al., 2020), and why *S. cynipsea* abounds around fresh dung all over Europe north of the Alps and there apparently outcompetes and ultimately relegates *S. neocynipsea* towards presumably marginal, high-altitude habitats (Pont & Meier, 2002). Such analyses would also provide further insights into patterns of interspecific gene flow across the species’ entire natural range, as well as into the role of local (e.g. longitudinal or latitudinal) adaptation in shaping genome-wide patterns of differentiation (e.g. Roy et al., 2018).

### Supplementary Information

Below is the link to the electronic supplementary material.

Supplementary material 1 (XLSX 71.6 kb)

Supplementary material 2 (PDF 64.6 kb)

Supplementary material 3 (ZIP 10.1 kb)

Supplementary material 4 (DOCX 427.2 kb)

Supplementary material 5 (XLSX 31.1 kb)

## Data Availability

All sequencing data have been deposited at the short-read archive (SRA; https://www.ncbi.nlm.nih.gov/sra) under the accession number PRJNA612154. Novel software is available at GitHub (https://github.com/nhmvienna/ABBABABA-4AF). A data matrix with allele frequencies for all SNPs and samples can be found on Data Dryad under the accession number 10.5061/dryad.fxpnvx0sm. Bioinformatic commands and custom scripts used in this study can be found at GitHub (https://github.com/capoony/SepsidABBABABA).
