# Supplementary material for: Geographic Variation in Genomic Signals of Admixture Between Two Closely Related European Sepsid Fly Species
Source: Evol Biol. 2023 Aug 25;50(4):395–412. doi: 10.1007/s11692-023-09612-5 (PMC10579158; doi:10.1007/s11692-023-09612-5)
Supplement: Supplementary file 2 — Supplementary material 2 (PDF 64.6 kb) [file 11692_2023_9612_MOESM2_ESM.pdf]

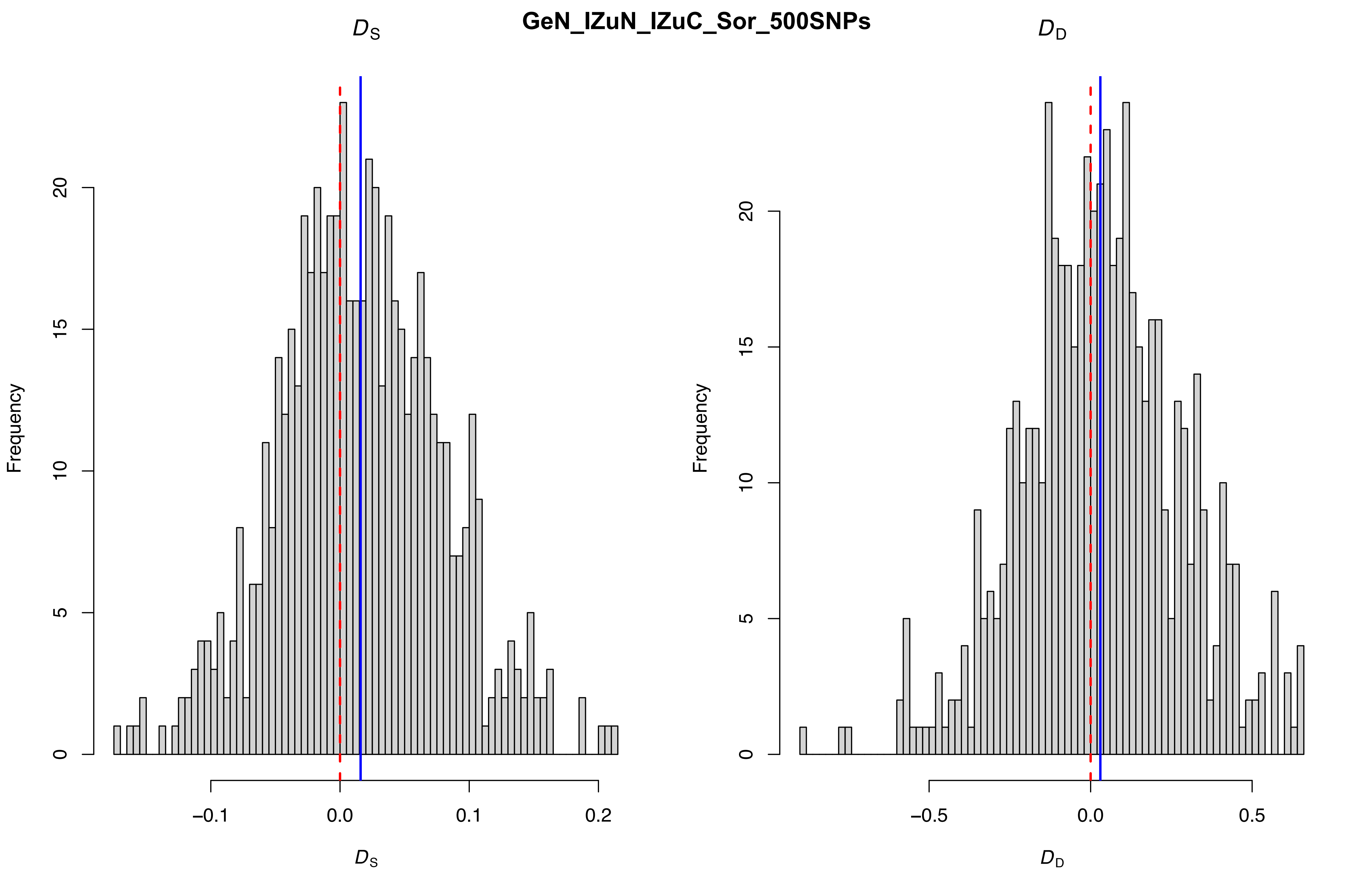

GeN_IZuN_IZuC_Sor_500SNPs
D_S
D_D
Frequency
0
5
10
15
20
−0.1
0.0
0.1
0.2
−0.5
0.5

GeN_IZuN_ZuC_Sor_500SNPs

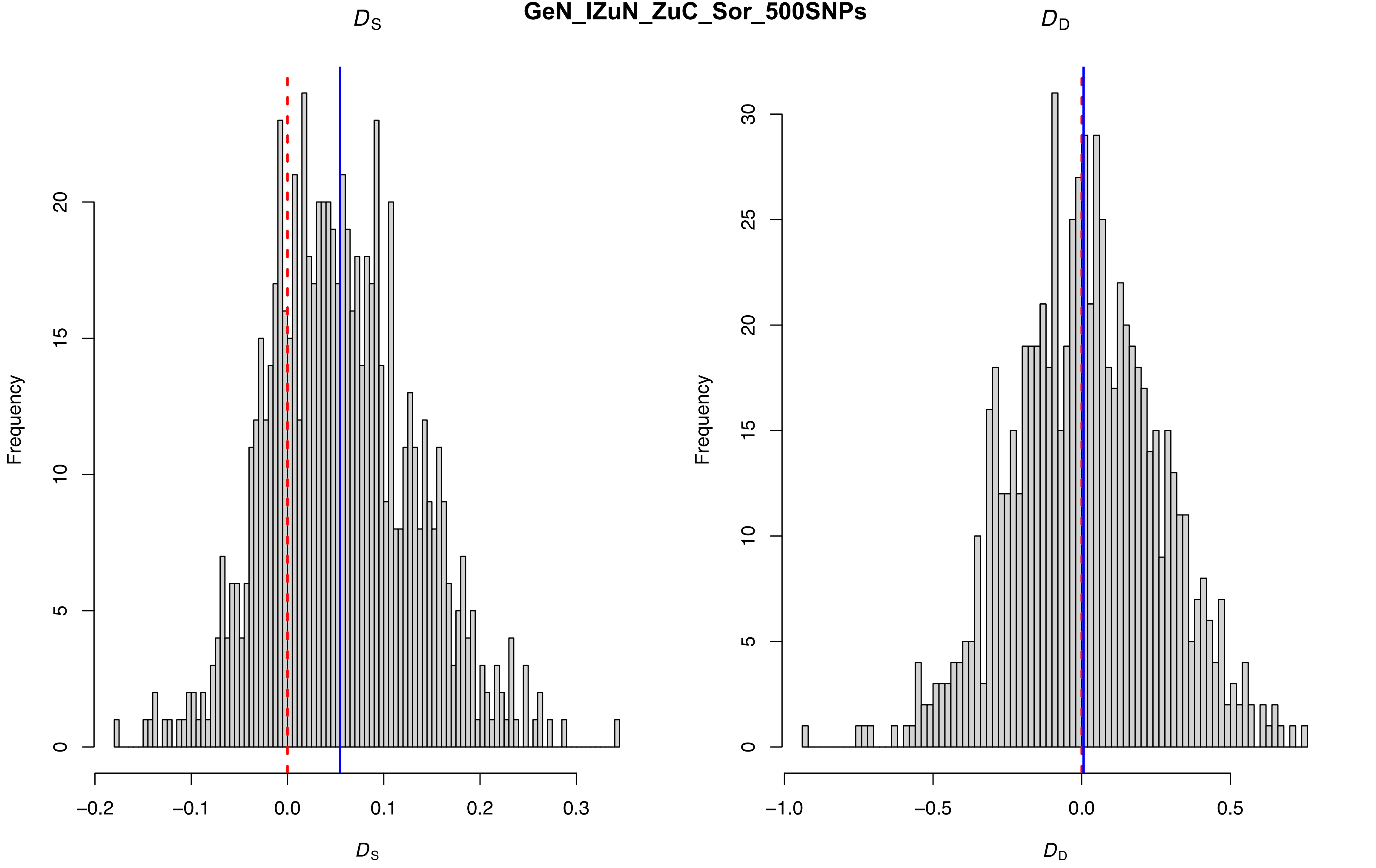

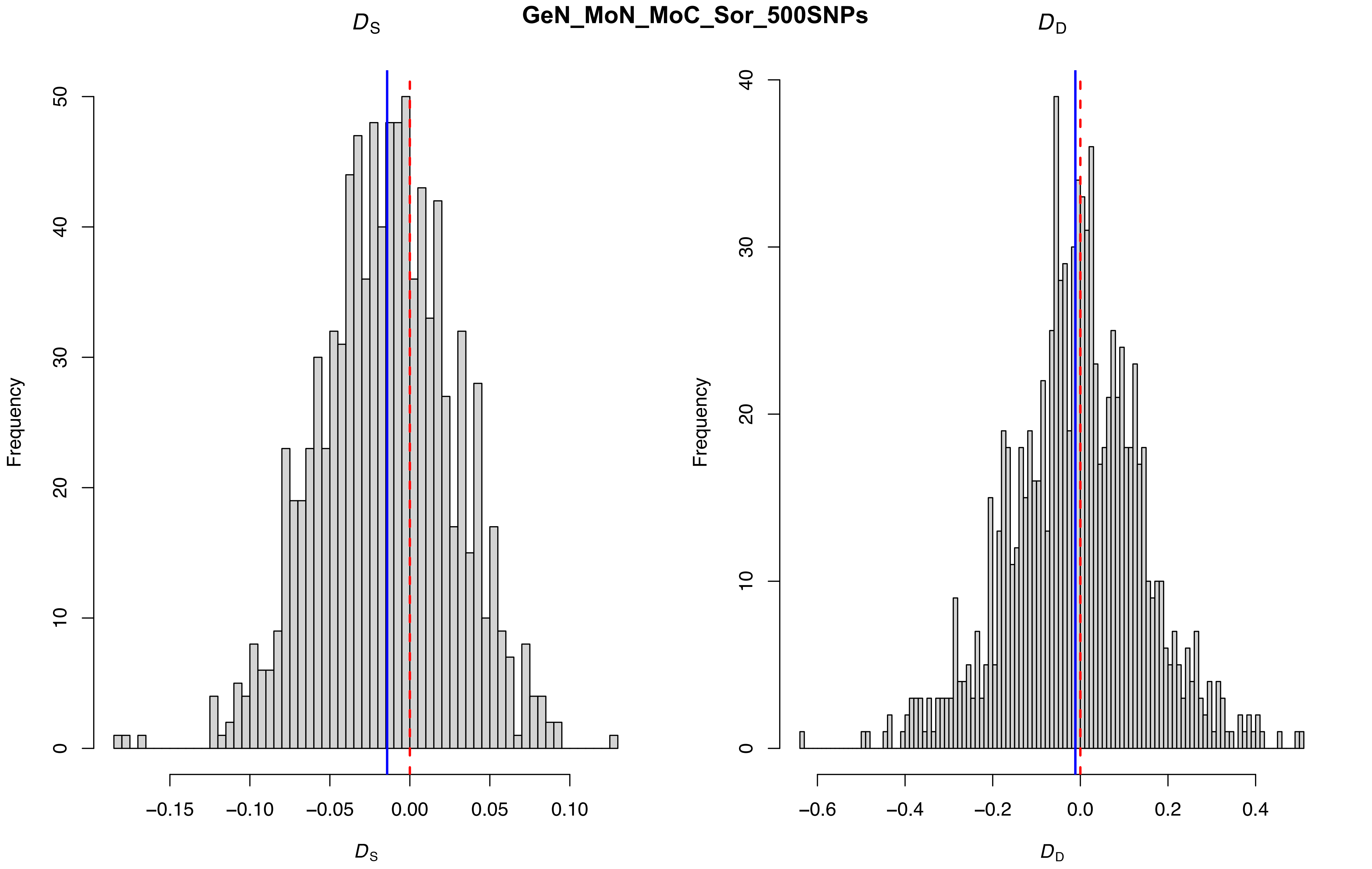

GeN_MoN_MoC_Sor_500SNPs
D_S
Frequency
0
10
20
30
40
50
−0.15
−0.10
−0.05
0.00
0.05
0.10
D_S
D_D
Frequency
0
10
20
30
40
−0.6
−0.4
−0.2
0.0
0.2
0.4
D_D

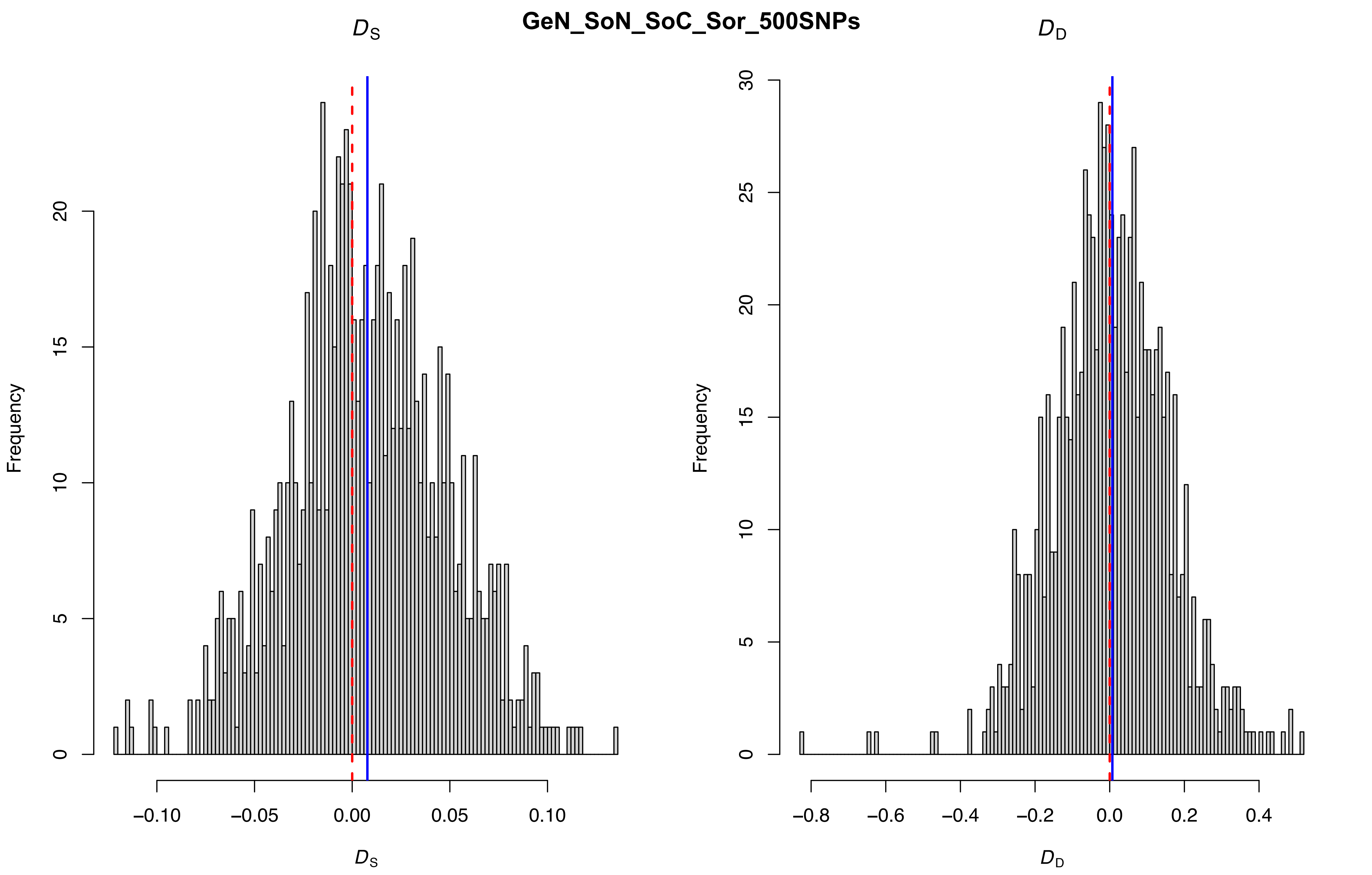

GeN_SoN_SoC_Sor_500SNPs
D_S
D_D
Frequency
0
5
10
15
20
25
30
−0.10
−0.05
0.00
0.05
0.10
−0.8
−0.6
−0.4
−0.2
0.0
0.2
0.4

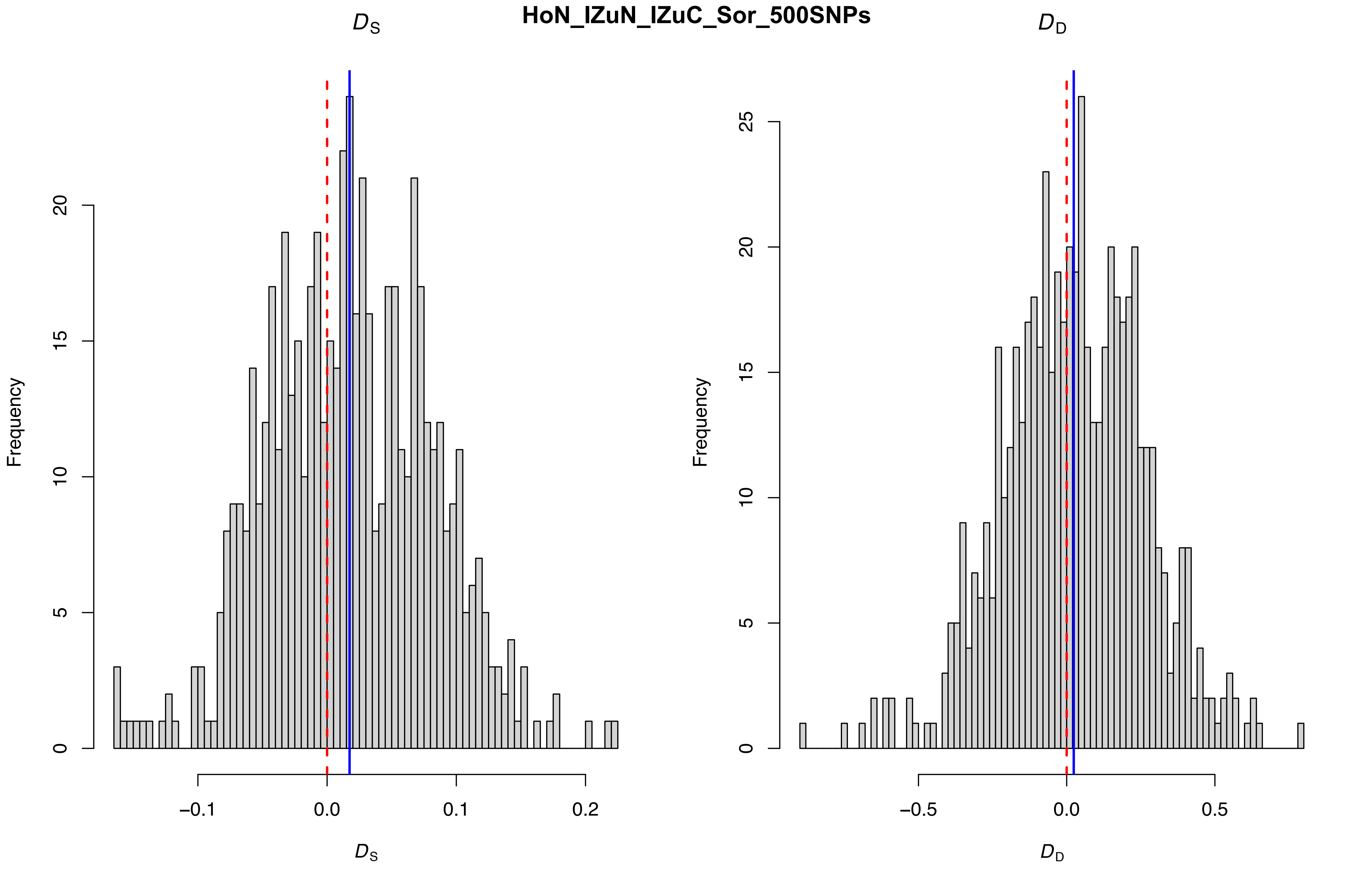
HoN_IZuN_IZuC_Sor_500SNPs
D_S
D_D
Frequency
0
5
10
15
20
25
−0.1
0.0
0.1
0.2
−0.5
0.5

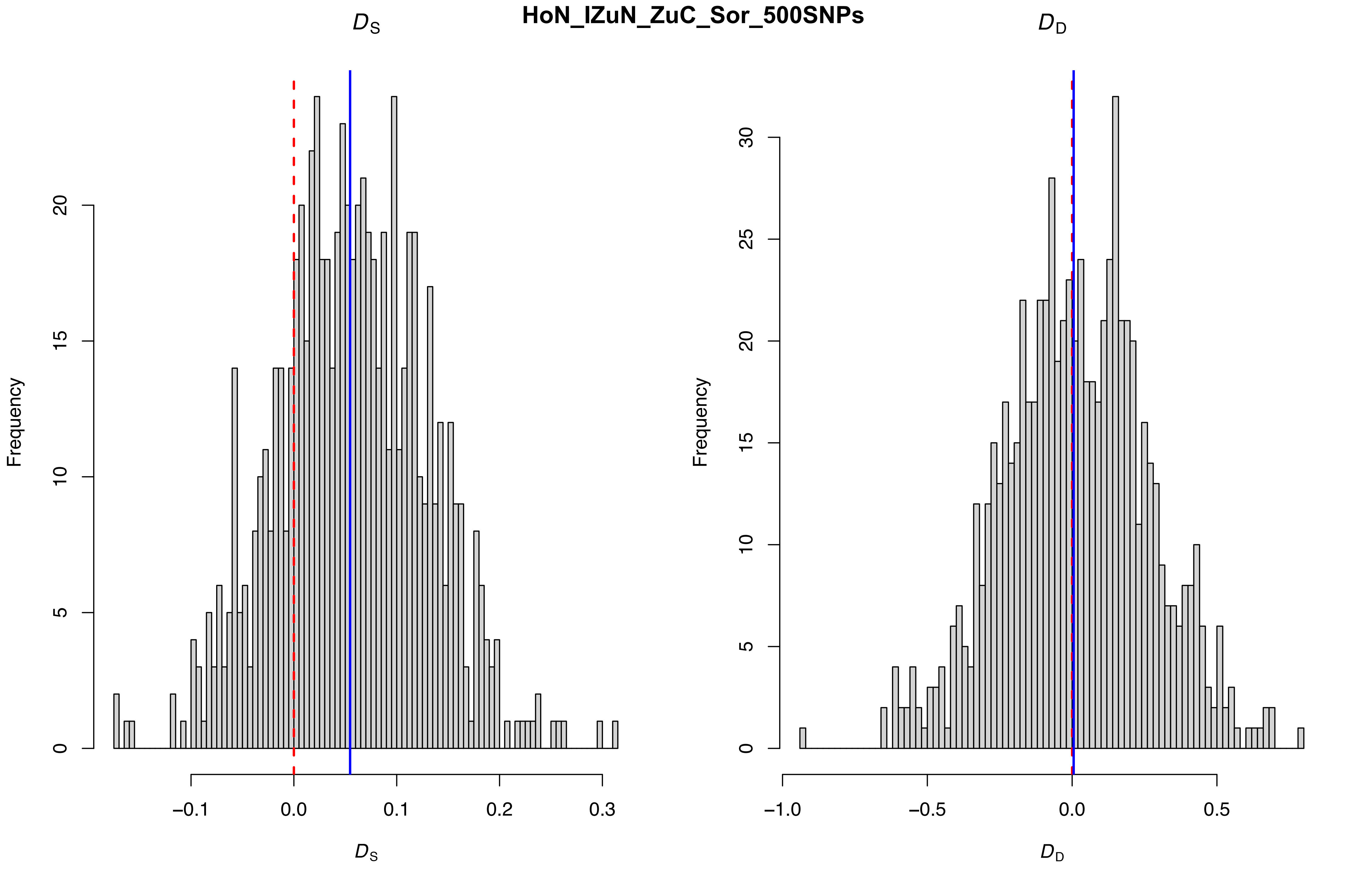

HoN_IZuN_ZuC_Sor_500SNPs
D_S
Frequency
0
5
10
15
20
−0.1
0.0
0.1
0.2
0.3
D_S
D_D
Frequency
0
5
10
15
20
25
30
−1.0
−0.5
0.0
0.5
D_D

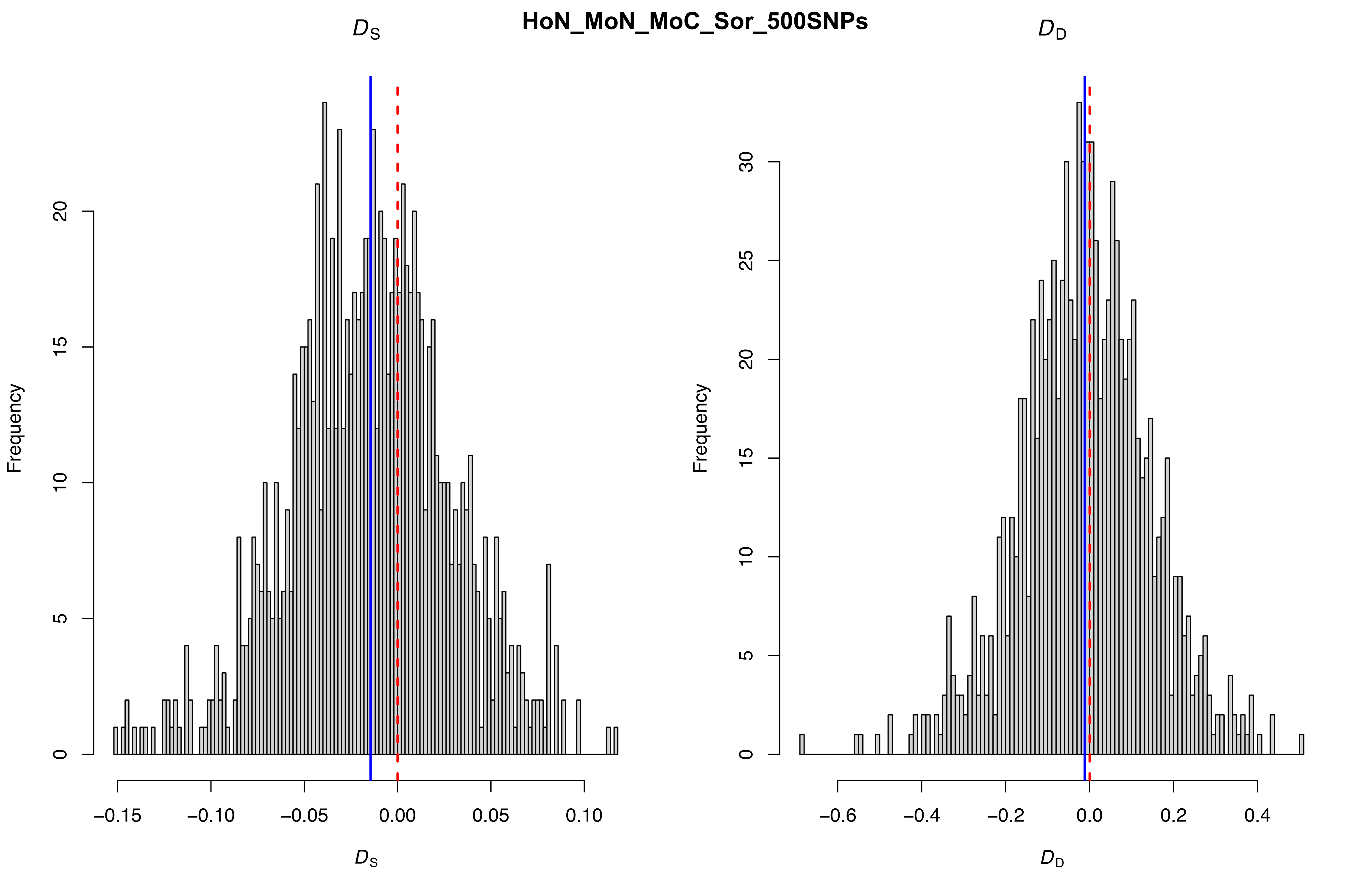

HoN_MoN_MoC_Sor_500SNPs
D_S
Frequency
0
5
10
15
20
−0.15
−0.10
−0.05
0.00
0.05
0.10
D_S
D_D
Frequency
0
5
10
15
20
25
30
−0.6
−0.4
−0.2
0.0
0.2
0.4
D_D

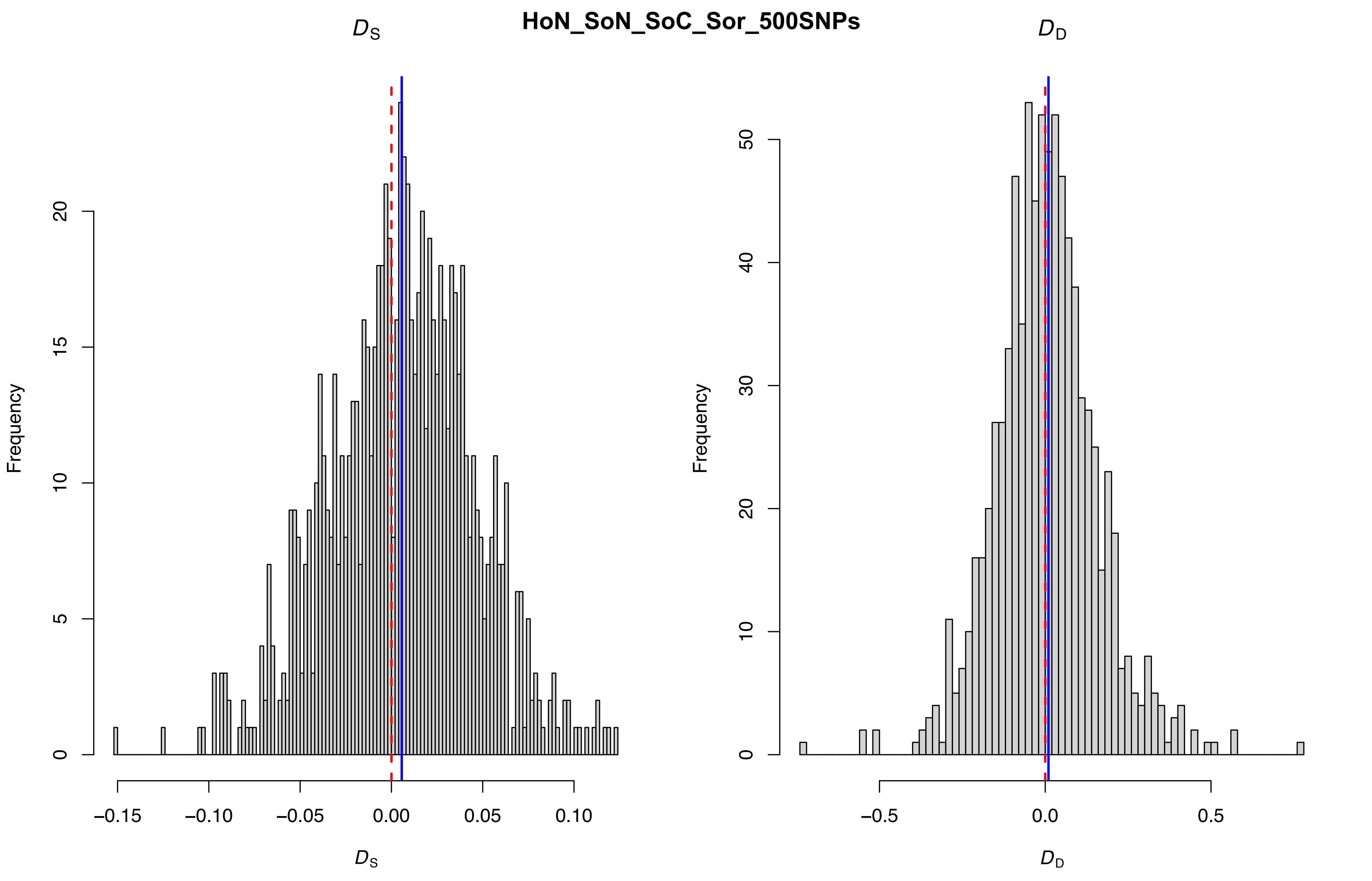
HoN_SoN_SoC_Sor_500SNPs
D_S
D_D
Frequency
Frequency
0
5
10
15
20
0
10
20
30
40
50
−0.15
−0.10
−0.05
0.00
0.05
0.10
−0.5
0.0
0.5
D_S
D_D

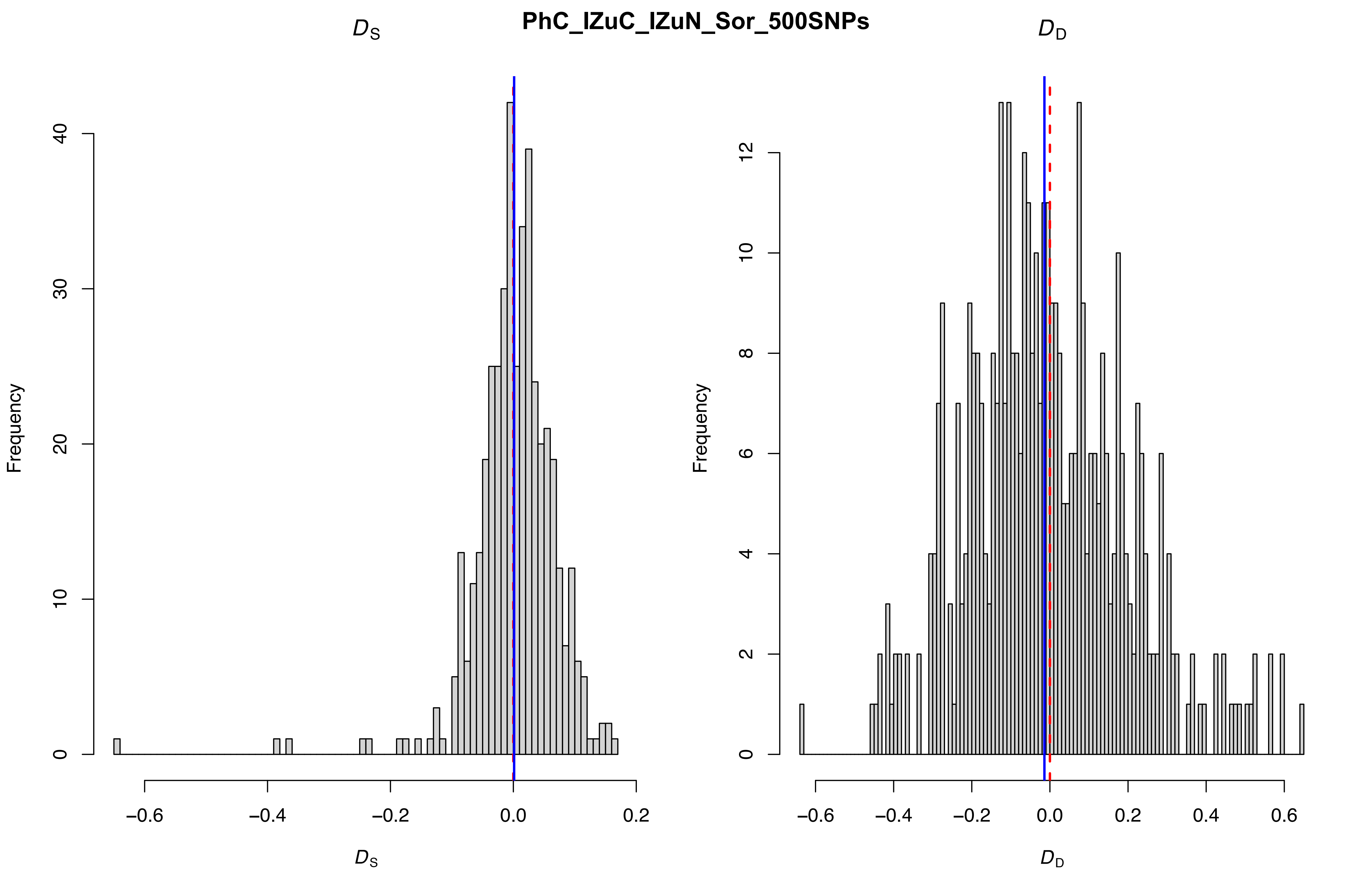

PhC_IZuC_IZuN_Sor_500SNPs
D_S
D_D
Frequency
0
10
20
30
40
−0.6
−0.4
−0.2
0.0
0.2
D_S
Frequency
0
2
4
6
8
10
12
−0.6
−0.4
−0.2
0.0
0.2
0.4
0.6
D_D

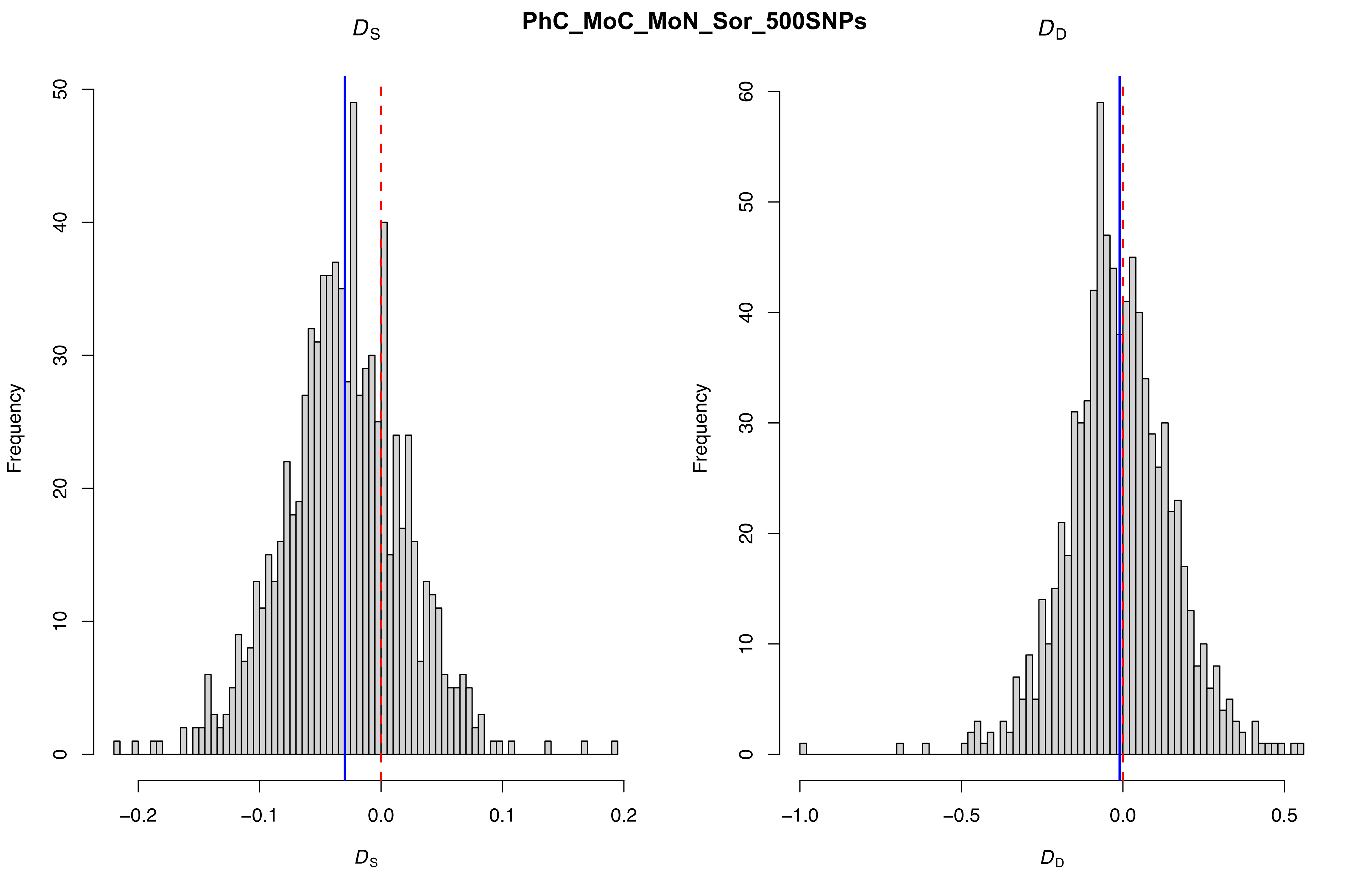

PhC_MoC_MoN_Sor_500SNPs
D_S
D_D
Frequency
0
10
20
30
40
50
60
−0.2
−0.1
0.0
0.1
0.2
−1.0
−0.5
0.5

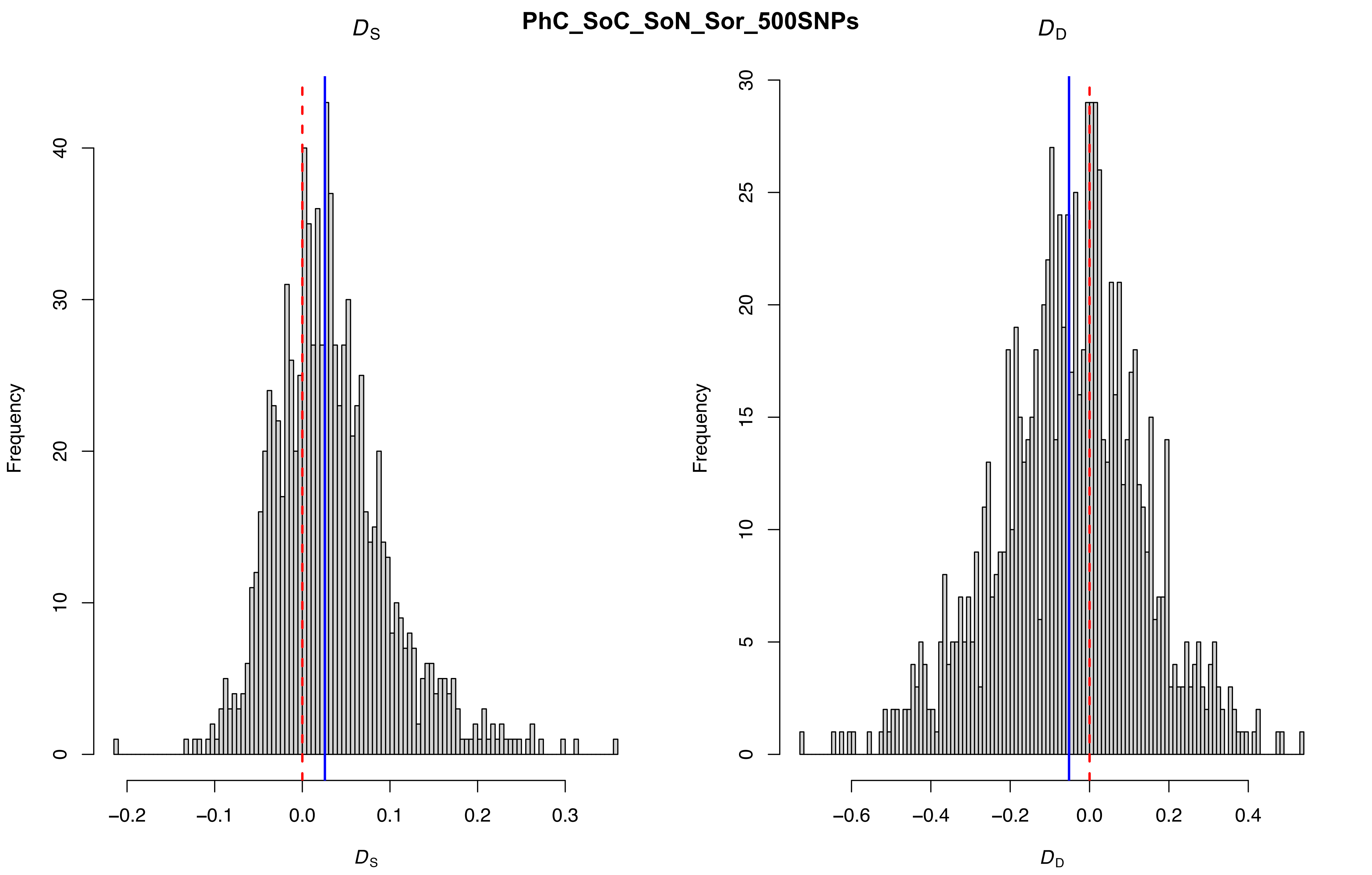

PhC_SoC_SoN_Sor_500SNPs
D_S
D_D
Frequency
0
10
20
30
40
−0.2
−0.1
0.0
0.1
0.2
0.3
0
5
10
15
20
25
30
−0.6
−0.4
−0.2
0.0
0.2
0.4

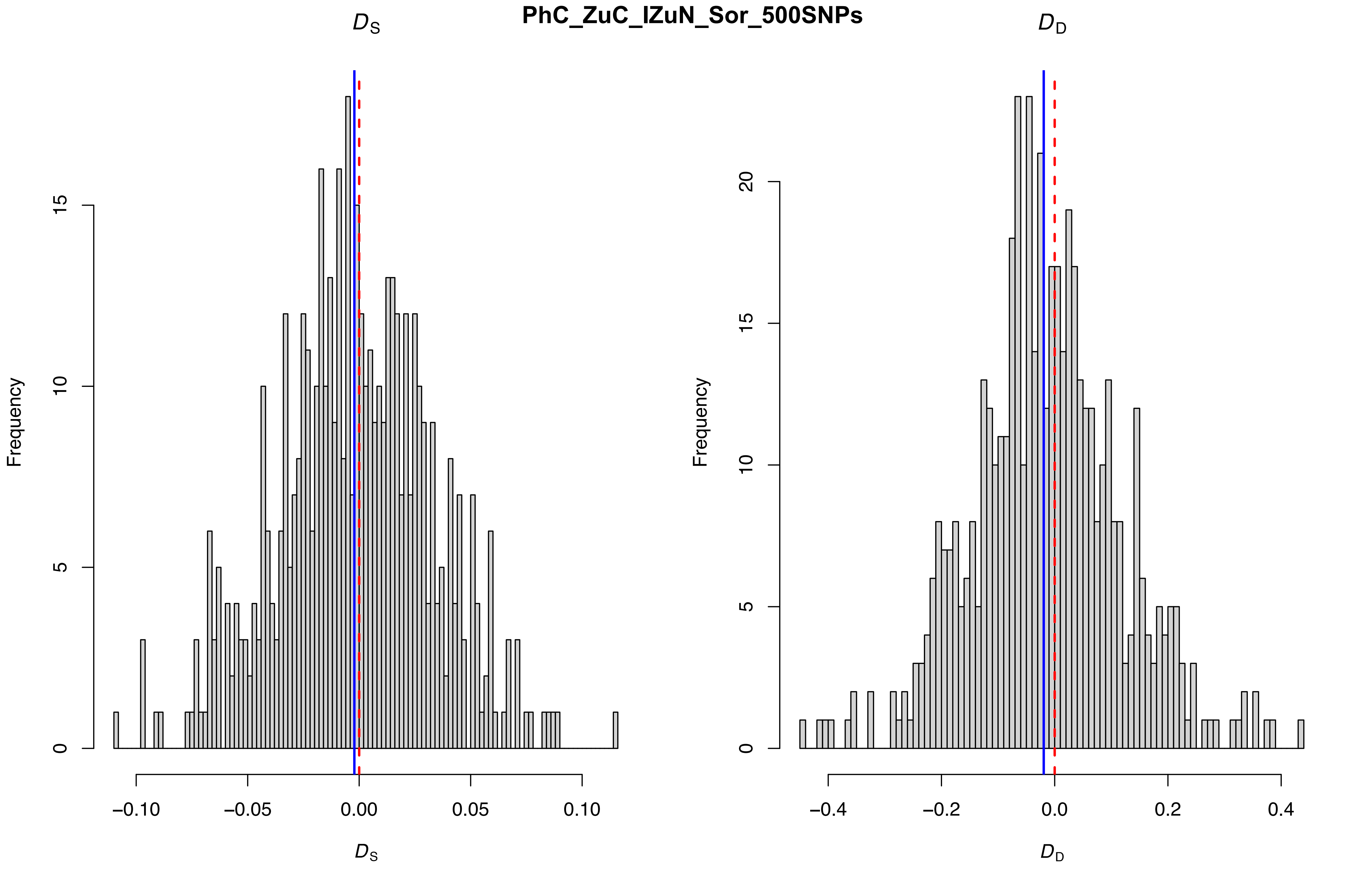
PhC_ZuC_IZuN_Sor_500SNPs
D_S
Frequency
0
5
10
15
−0.10
−0.05
0.00
0.05
0.10
D_S
D_D
Frequency
0
5
10
15
20
−0.4
−0.2
0.0
0.2
0.4
D_D

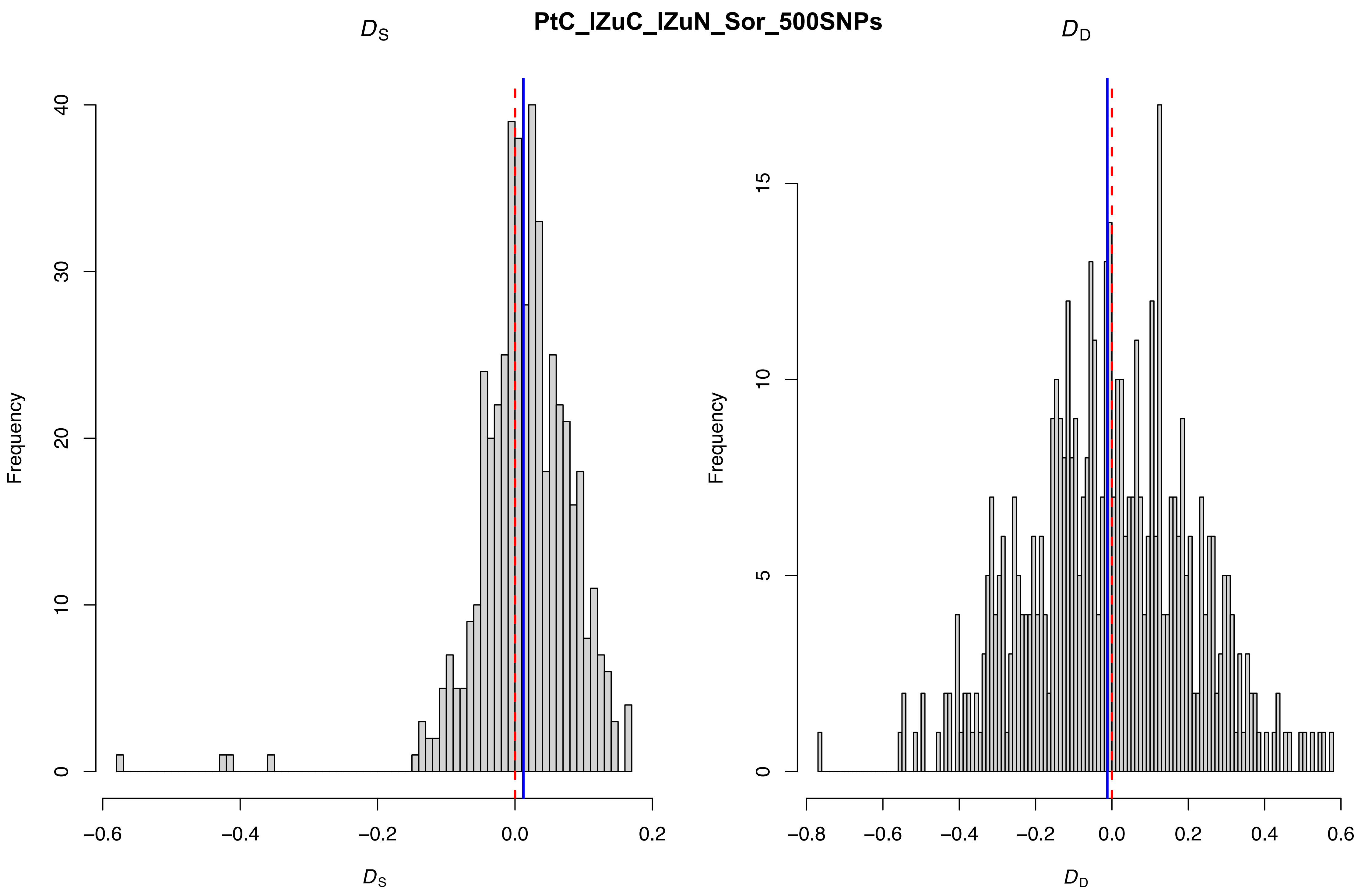
PtC_IZuC_IZuN_Sor_500SNPs
D_S
D_D
Frequency
0
10
20
30
40
−0.6
−0.4
−0.2
0.0
0.2
D_S
Frequency
0
5
10
15
−0.8
−0.6
−0.4
−0.2
0.0
0.2
0.4
0.6
D_D

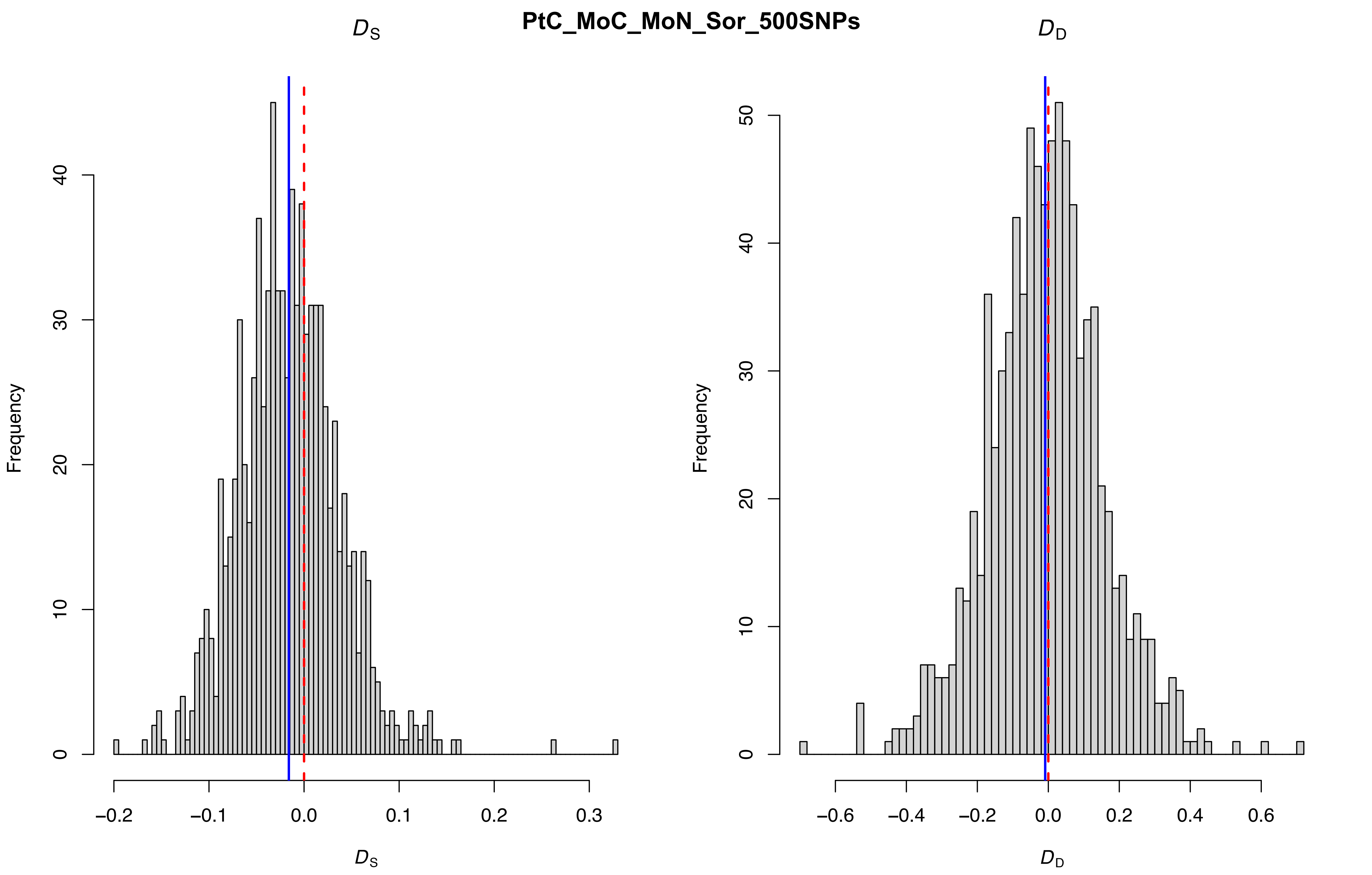

PtC_MoC_MoN_Sor_500SNPs
D_S
D_D
Frequency
0
10
20
30
40
50
−0.2
−0.1
0.0
0.1
0.2
0.3
−0.6
−0.4
−0.2
0.0
0.2
0.4
0.6

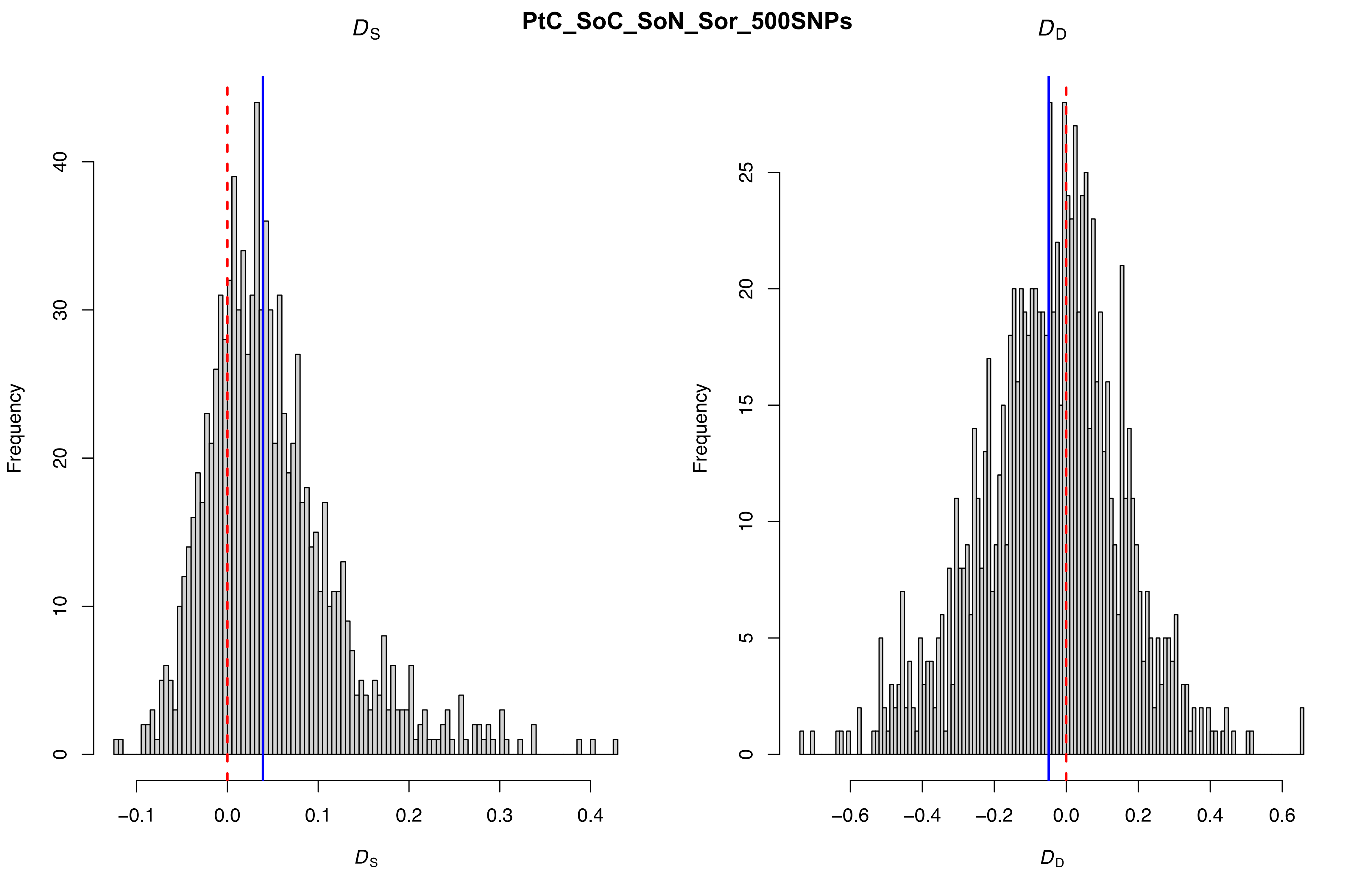

PtC_SoC_SoN_Sor_500SNPs
D_S
D_D
Frequency
0
10
20
30
40
−0.1
0.0
0.1
0.2
0.3
0.4
Frequency
0
5
10
15
20
25
−0.6
−0.4
−0.2
0.0
0.2
0.4
0.6
D_S
D_D

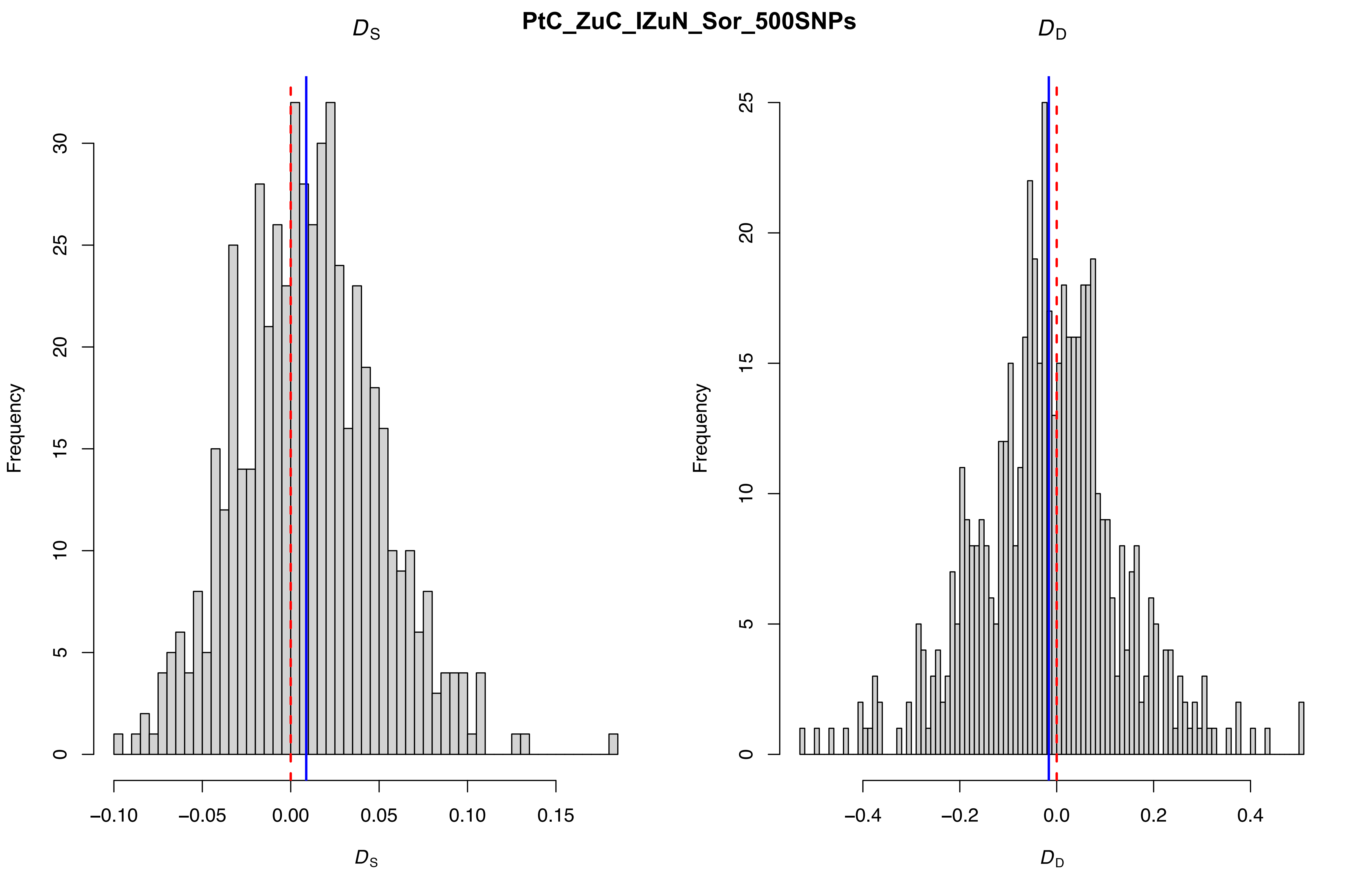
PtC_ZuC_IZuN_Sor_500SNPs
D_S
D_D
Frequency
Frequency
0
5
10
15
20
25
30
0
5
10
15
20
25
−0.10
−0.05
0.00
0.05
0.10
0.15
−0.4
−0.2
0.0
0.2
0.4
D_S
D_D
