# Supplementary material for: Geographic Variation in Genomic Signals of Admixture Between Two Closely Related European Sepsid Fly Species
Source: Evol Biol. 2023 Aug 25;50(4):395–412. doi: 10.1007/s11692-023-09612-5 (PMC10579158; doi:10.1007/s11692-023-09612-5)
Supplement: Supplementary file 4 — Supplementary material 4 (DOCX 427.2 kb) [file 11692_2023_9612_MOESM4_ESM.docx]

# Geographic variation in genomic signals of admixture between two closely related European sepsid fly species

Athene Giesen^1^, Wolf U. Blanckenhorn^1^, Martin A. Schäfer^1^, Kentaro K. Shimizu^1^, Rie Shimizu-Inatsugi^1^, Bernhard Misof^2^, Oliver Niehuis^3^, Lars Podsiadlowski^2^, Heidi E. L. Lischer^1,4^, Simon Aeschbacher^1^, Martin Kapun^1,5,6^

^1^Department of Evolutionary Biology and Environmental Studies, University of Zürich, Switzerland

^2^Zoological Research Museum Alexander Koenig, Bonn, Germany

^3^Department of Evolutionary Biology and Ecology, Institute of Biology I (Zoology), Albert Ludwig University, Freiburg, Germany

^4^Interfaculty Bioinformatics Unit, University of Bern, Switzerland

^5^Division of Cell & Developmental Biology, Medical University of Vienna, Austria

^6^Natural History Museum Vienna, Vienna, Austria

**Supplementary Material**

**Supplementary Methods and Results**

Supplementary Text S1

*Reduction of genetic diversity in laboratory populations*

We expected the genetic diversity in the laboratory population of *S. cynipsea* derived from single wild-caught females from the Zürich site to be strongly reduced relative to that of field-caught flies from the same site. While we did observe significantly lower levels of expected heterozygosity (Nei 1987) in laboratory than field-caught populations (Mann Whitney U test, *p* < 0.001; Supplementary Figure S5), reductions in heterozygosity were much lower than expected under strong inbreeding. Our laboratory populations were therefore not highly inbred, so we decided to treat the sequencing data from laboratory and natural populations equally in all our analyses.

To interpret the decrease in genetic diversity that we observed in our laboratory populations relative to field-caught flies, we adopted a simple population genetic model for the decay of the expected heterozygosity *π* (Nei 1987). Specifically, we assumed that a single wild-caught female mated to *N_m_* males was brought into the laboratory to initiate a panmictic population of effective size *N_e_*, and that this population was then maintained for *n* generations. According to Eq. (3.122) of Ewens (2004, p. 123), and given a single founding female, we approximated the effective population size during the founder event at generation 0by $4N_{m}/(1+N_{m})$. The expected heterozygosity at generation *t* is then given by

$\pi_{t}=\left( 1-\frac{1}{2\times4N_{m}/(1+N_{m})} \right)\left( 1-\frac{1}{2N_{e}} \right)^{t-1}\pi_{0,}$ (Eq. S1)

where *π*_0_ is the expected heterozygosity in the natural population. By substituting the expected heterozygosity computed from allele frequencies in field-caught and laboratory populations after *t* = 30 generations *π*_0_ and *π*_30_ respectively, we explored which combinations of *N_m_* and *N_e_* would explain the observed reduction in *π* (Supplementary Figure S6). We limited *N_e_* to 100, our upper estimate for the census size of the laboratory populations. We set *π*_0_ = 0.05% and *π*_30_ = 0.033% according to our estimates. To explain this 34% reduction in heterozygosity over 30 generations conditional on a single founding female and assuming that it mated a single male (*N_m_* = 1), an effective size of *N_e_* = 100 of the subsequent laboratory population is needed (Supplementary Figure S6). If instead we assume the female had mated a perhaps unrealistically high number of 100 males prior to being introduced to the lab, we would still require an effective size of no lower than *N_e_* = 50 to explain the observed reduction in heterozygosity (Supplementary Figure S6). These results thus suggest that the initial founder event and successive breeding in the laboratory induced at most moderate inbreeding.

Supplementary Text S2

*Exploratory analyses of microsatellite data to inform demographic inference*

Interpretation of the ABBA-BABA test for admixture (see Materials and Methods) requires prior knowledge about the evolutionary relationship among taxa or populations included in the test. To gain this knowledge for our 8 European *S. cynispsea* and *S. neocynipsea* populations chosen from previously published data by Baur *et al.* (2020), we compared alternative demographic scenarios and estimated demographic parameters using ABC (Supplementary Text S3; Model comparison and parameter inference using Approximate Bayesian Computation). To limit the set of all possible population tree topologies to be explored in these ABC demographic analyses, we first identified a smaller set of candidate topologies supported by the data. Specifically, we inferred a neighbour-joining (NJ) tree using the *R* package *ape* (Paradis *et al.* 2004) from linearised pairwise $F_{ST}$ computed at all 9 microsatellites with the *hierfstat* package (Goudet 2005) in *R* from the dataset by Baur et al. (2020). The topology of the resulting NJ population tree confirmed the basal split between European *S. cynispsea* and *S. neocynipsea* (Supplementary Figure S1). For *S. cynispsea*, the NJ tree further suggested that the three Swiss populations from Geschinen (GeC), Sörenberg (SoC), and Zürich (ZuC) are more closely related to each other than any of them is to the populations from Pehka (PhC) and Petroia (PtC). However, the microsatellite data provided little information to resolve the relationships among the three Swiss populations (Supplementary Figure S1). The NJ tree also suggested that PhC and PtC find no common ancestor before they merge with the ancestor of the three Swiss populations, and that the Italian PtC population is genetically less diverged from the three Swiss populations (jointly denoted by ChC in the following; Figure 1) than is the Estonian PhC population. While this topology, ((ChC, PtC), PhC), is consistent with increasing genetic isolation by geographic distance (Italy being closer to the Swiss Alps than Estonia), we did not yet exclude the alternative topology in which PhC and PtC find a common ancestor before they merge with the ancestor of ChC, i.e. (ChC, (PhC, PtC)).

For *S. neocynipsea*, the NJ tree suggested that the populations from Sörenberg (SoN) and Zürich (ZuN) find a common ancestor before merging with the population from Geschinen (GeN; Supplementary Figure S1). This result is surprising given that SoN is geographically closer to GeN than to ZuN. We therefore kept all three possible sub-topologies in the initial set, including ((GeN, SoN), ZuN). In summary, we defined a set of twelve species-specific initial candidate topologies, nine for *S. cynipsea* and three for *S. neocynipsea* (Supplementary Table S2; Supplementary Figure S2). This set includes the topology suggested by the microsatellite NJ tree, as well as alternative (sub-)topologies to account for the sources of uncertainty discussed above. We subjected demographic scenarios derived from these candidate topologies to a first round of ABC model comparison to identify the best-performing scenario(s) for each species (see Supplementary Text S3 and the DIYABC-specific header files “header_Scyn.txt” and “header_Sneo.txt” in GiesenEtAl_EB_SupplementaryMaterial_HeaderFiles.zip for the demographic models and prior distributions).

Supplementary Text S3

*Model comparison and parameter inference using Approximate Bayesian Computation*

To rate the twelve candidate population topologies identified in Supplementary Text S2 in terms of their support by the microsatellite data, and to estimate demographic parameters under the best-supported models, we used Approximate Bayesian Computation (ABC; Beaumont 2019) as implemented in DIYABC v.2.0.1 (Cornuet et al. 2014). The program implements coalescent simulations and performs model comparison and parameter estimation under demographic scenarios specified by the user. We augmented the candidate population topologies discussed in Supplementary Text S2 to respective demographic scenarios as follows. First, we assigned to the $i$*-*th population split backward in time a split time $t_{i}$, measured in generations from the time of sampling. Second, we assigned to each branch $b$ in the topology a constant effective population size $N_{b}$, where $b$ is a string encoding the set of leaf nodes (sampled populations) subtending the respective branch. For instance, $N_{123}$ refers to the effective size of the population along the branch that will give rise to the sampled populations 1, 2, and 3.

We then performed two rounds of ABC model comparison. The aim of the first round was to rank the nine and three species-specific demographic scenarios derived from the respective candidate population topologies for *S. cynipsea* and *S. neocynipsea,* respectively*,* by their ABC posterior support (Supplementary Figure S2). In the second round, we selected for each species the best-performing demographic scenario(s) established in round 1, and then compared four joint scenarios resulting from all possible combinations of species-specific scenarios in terms of their ABC posterior support. In these joint demographic scenarios, we denoted the species split time by $t_{cn}$. In both rounds of ABC model comparison, we simulated one million datasets per demographic scenario, with input values for the demographic parameters sampled from prior distributions given in the header files of the corresponding DIYABC analyses (“header_Scyn.txt” and “header_SNeo.txt” for the first round and “header_Full.txt” for the second round; see GiesenEtAl_EB_SupplementaryMaterial_HeaderFiles.zip). To match the empirical data from Baur *et al.* (2020), we simulated 9 microsatellite loci for each dataset under the generalised stepwise mutation model (GSM).

To compare simulated and observed data, we used three one-sample summary statistics (i.e. statistics computed separately for each population) and seven two-sample summary statistics (i.e. statistics computed for each possible two-population set of individuals). The one-sample statistics comprised the mean number of alleles across microsatellite loci, the mean of Nei’s (1978) gene diversity across loci, and the mean allele size variance across loci. Three of the two-sample statistics were analogous to the three one-sample statistics. The remaining four two-sample statistics were the shared allelic distance between the two population samples (Chakraborty and Jin 1993), the mean index of classification Rannala and Mountain (1997), the genetic population differentiation in terms of Weir and Cockerham’s (1984) $F_{\mathrm{ST}}$, and the squared difference in mean allele lengths between the two populations, ${(\delta\mu)}^{2}$, as proposed by Goldstein et al. (1995).

We compared competing demographic scenarios in terms of their posterior probabilities. To estimate these probabilities, we used the weighted multinomial logistic regression approach implemented in DIYABC and originally proposed by Fagundes et al. (2007) and Beaumont (2008). To reduce the dimensionality of the regression problem, we chose the DIYABC option of replacing the full set of summary statistics by the first x components of a linear discriminant analysis (LDA) of the log-transformed summary statistics. In this LDA, only the 1 percent simulated datasets closest in terms of Euclidean distance to the observed data were used. We considered the difference in posterior probability point estimates for two scenarios significant if the respective 95% confidence intervals did not overlap.

In our first round of ABC model selection (i.e. comparison of species-specific demographic scenarios), we kept for *S. cynpisea* the top four and for *S. neocynipsea* the single top demographic scenario(s) with the highest posterior probability estimate(s). We then combined these 4x1 topologies from round one into four combined scenarios that include both species to perform a second round of ABC model selection (i.e., the comparison of joint demographic scenarios shown in Supplementary Figure S2C). For the demographic model that performed best, we used DIYABC to infer posterior distributions for all model parameters using the default settings of DIYABC.

Given the best-performing joint demographic scenario, we derived from this scenario pruned scenarios that only included three focal populations and one outgroup population to mirror the sets of populations used in the respective ABBA-BABA tests for gene flow (see Materials and Methods for details). To these pruned demographic scenarios, we added two admixture proportions, $m_{1 \leftrightarrow3}$ and $m_{2 \leftrightarrow3}$, to reflect single pulses of bidirectional interspecific gene flow in allopatry and sympatry, respectively (Supplementary Figure S3; Methods in main text). Depending on whether we tested a scenario with stronger sympatric or allopatric gene flow, we set the conditions $m_{1 \leftrightarrow3}$ < $m_{2 \leftrightarrow3}$ (assuming increased local gene flow; see Scenario 2A in Figure S3) or $m_{1 \leftrightarrow3}$ > $m_{2 \leftrightarrow3}$ (assuming reduced local gene flow; see Scenario 2B in Figure S3). We compared the fit of the resulting demographic scenarios (and their counterparts without gene flow) to the corresponding microsatellite data from Baur et al. (2020) using the same ABC scheme as described above. We also inferred the demographic parameters with ABC as described above; for the bidirectional admixture proportions we used a uniform prior ranging from 0 to 0.5 and for the time of the admixture pulses we assumed a log-uniform prior ranging from 0 to 1000 generations (see also DIYABC-specific header files “header_PhC-SoC-SoN.txt”, “header_PtC-SoC-SoN.txt”, “header_PhC-ZuC-ZuN.txt” and “header_PtC-ZuC-ZuN.txt” for the four different analyses in GiesenEtAl_EB_SupplementaryMaterial_HeaderFiles.zip for further information).

Supplementary Text S4

Overview of ABBA-BABA test statistics

In the original ABBA-BABA test, Green *et al.* (2010) computed the *D*-statistic across *n* bases as

$$D_{G}=\frac{\sum_{i=1}^{n} \left[ C_{ABBA}\left( i \right)-C_{BABA}(i) \right]}{\sum_{i=1}^{n} \left[ C_{ABBA}(i)+C_{BABA}(i) \right]},$$

where *C*_ABBA_(*i*) and *C*_BABA_(*i*) are indicator variables taking a value of 0 or 1 depending on whether an ABBA or BABA configuration is observed at base *i*. This version of the test was developed for aligned sequence data from four haploid individuals (or, equivalently, diploid individuals made pseudo-haploid by random sampling of alleles). To extend the scope of the ABBA-BABA test to allele frequencies at a set of SNPs, Durand *et al.* (2011) proposed the modified *D* statistic

$$D_{D}=\frac{\sum_{i=1}^{n} \left[ \left( 1-\hat{p}_{i1} \right)\hat{p}_{i2}\hat{p}_{i3}(1-\hat{p}_{i4})-\hat{p}_{i1}(1-\hat{p}_{i2})\hat{p}_{i3}(1-\hat{p}_{i4}) \right]}{\sum_{i=1}^{n} \left[ \left( 1-\hat{p}_{i1} \right)\hat{p}_{i2}\hat{p}_{i3}\left( 1-\hat{p}_{i4} \right)+\hat{p}_{i1}(1-\hat{p}_{i2})\hat{p}_{i3}(1-\hat{p}_{i4}) \right]},$$

where $\hat{p}_{ij}$ is the estimated frequency of the derived allele *B* at SNP *i* in population (species) *j*. An excess of the ABBA allele pattern thus results in a positive *D_G_* or *D_D_*, whereas an excess of the BABA allele pattern results in a negative *D_G_* or *D_D_*.

Recently, Soraggi *et al.* (2018) proposed an extension of the original ABBA-BABA test that accommodates data at *M* genomic sites from multiple individuals per population sequenced at varying depth. Soraggi *et al.* (2018) expressed the probabilities of observing ABBA and BABA patterns in terms of their expected values with respect to the population allele-frequency distributions,

$$Pr\left( {ABBA}_{i} \right)=E\left[ {\left( 1-p_{i1} \right)p_{i2}p_{i3}\left( 1-p_{i4} \right)+p}_{i1}\left( 1-p_{i2} \right)\left( 1-p_{i3} \right)p_{i4} \right],$$

and

$$Pr\left( {BABA}_{i} \right)=E\left[ {\left( 1-p_{i1} \right)p_{i2}\left( 1-p_{i3} \right)p_{i4}+p}_{i1}\left( 1-p_{i2} \right)p_{i3}\left( 1-p_{i4} \right) \right].$$

Under the null hypothesis of no gene flow, the probabilities Pr(ABBA*_i_*) and Pr(BABA_i_) are equal, i.e.

$$H_{0}: Pr\left( {BABA}_{i} \right)-Pr\left( {ABBA}_{i} \right)=E\left[ \left( p_{i1}-p_{i2} \right)\left( p_{i3}-p_{i4} \right) \right]=0 for all i=1,\ldots,M.$$

Equivalent to the approach of Durand *et al.* (2011), Soraggi *et al.* (2018) normalized by

$$Pr\left( {BABA}_{i} \right)+Pr\left( {ABBA}_{i} \right)=E\left[ \left( p_{i1}+p_{i2}-2p_{i1}p_{i2} \right)\left( p_{i3}+p_{i4}-2p_{i3}p_{i4} \right) \right].$$

For empirical allele frequencies $\hat{p}_{ij}$ as unbiased estimators of $p_{ij} (j=1,\ldots,4)$, Soraggi *et al.* (2018) defined their extended *D*-statistic as

$$D_{S}=\frac{\sum_{i=1}^{n} \left[ \left( \hat{p}_{i1}-\hat{p}_{i2} \right)\left( \hat{p}_{i3}-\hat{p}_{i4} \right) \right]}{\sum_{i=1}^{n} \left[ \left( \hat{p}_{i1}+\hat{p}_{i2}-2\hat{p}_{i1}\hat{p}_{i2} \right)\left( \hat{p}_{i3}+\hat{p}_{i4}-2\hat{p}_{i3}\hat{p}_{i4} \right) \right]},$$

and showed that the distribution of *D*_S_ converges to a Gaussian distribution under *H*_0_. Note that, due to how *H*_0_ was formulated, *D*_S_ has the opposite sign of *D*_D_ and *D*_G_, i.e. an excess (deficiency) of the BABA (ABBA) allele pattern implies *D*_S_ > 0, and *vice versa.* We followed Soraggi *et al.* (2018) in restricting our analyses to informative sites only, i.e. sites at which samples from P1 and P2, or P3 and P4 (cf. above and Fig. 2) are not fixed for the same allele. This restriction avoids calculating *D*_D_ and *D*_S_ in cases in which these statistics are not defined because their denominators are zero.

We further note that *D*_S_ differs from *D*_D_ in that *D*_S_ accounts for the expected frequencies of both ABBA and BAAB in Pr(ABBA*_i_*), and for both BABA and ABAB in Pr(BABA*_i_*). In other words, the expectations integrate over the allelic state of the outgroup. We suspect that this difference implies differences in the distributions of *D*_D_ and *D*_S_ as well as in the rates at which the distributions converge to a Gaussian distribution as *n* increases. While a detailed investigation of these aspects is beyond the scope of this paper, we anticipate that they affect the robustness of the statistics. Indeed, the histograms of the two statistics looked different for many of the test configurations we explored (Supplementary Figures S4; Table 2). Overall, the distribution of *D*_S_ tended to have shorter tails and more probability mass in the shoulders compared to the distribution of *D*_D_. These observations might explain why, for some test configurations, the test based on *D*_S_ was significant, but the one based on *D*_D_ was not (Table 2).

*Implementation of ABBA-BABA tests based on allele frequency data*

The two approaches to calculating *D*-statistics by Green *et al.* (2010) and Soraggi *et al.* (2018) are implemented in the software ANGSD (Korneliussen, Albrechtsen & Nielsen, 2014). While ANGSD integrates over site-specific genotype likelihoods when calculating *D*, it currently does not support pool-sequencing data (but see Deitz *et al.* 2016). Moreover, ANGSD does not provide the possibility to use a predefined set of high-confidence SNPs, since variants are called based on the provided samples as part of the analysis. We therefore implemented the calculation of the *D*-statistics from allele frequency data by Durand *et al.* (2011) and by Soraggi *et al.* (2018) in a Python script available from <https://github.com/capoony/ABBABABA-4AF>. Our script calculates *D* both across the entire genome as well as within non-overlapping genomic windows defined to all either contain the same number of SNPs or to be equal in sequence length. To test for significant deviations of *D* from 0, our script also calculates *z*-scores based on jackknifing following the approach suggested by Busing *et al.* (1999). When using windows with equal numbers of SNPs, we perform a blocked, even *m*-delete jackknife procedure, where *m* is the group size of observations removed from the sample for jackknifing. This differs from the method implemented in ANGSD. ANGSD uses an uneven *m*-delete procedure because it uses windows of equal sequence length, which may contain unequal numbers of SNPs. We removed *m* = 1 window (block) at a time to obtain standard deviations and calculate *z*-scores following Green *et al.* (2010). We adopted the commonly used significance threshold of |*z*| > 3 (Reich *et al.,* 2011; Jeong *et al.,* 2016; Novikova *et al.,* 2016).

In contrast to Soraggi et al. (2018), who computed the *D*-statistic for human genomic data in windows of 5 million base pairs, we chose equally sized blocks of 500 SNPs for the calculation of *D* given that sepid genomes are ~20-fold smaller than the human genome. Keeping the number of SNPs constant in each block results in similar variances across blocks and facilitates the estimation of variances using the even *m*-block jackknife method (Busing et al. 1999). However, to test for the robustness of our approach, we repeated the analyses with different block sizes (based on 100, 500 and 100 SNPs) and sequence length (100,000 and 200,000 bp) and found highly significant correlations among the *z*-scores of the different analyses (see Supplementary Figure S7). This result indicates that our ABBA-BABA results are not influenced by the choice of window-size and the type of blocking.

**Supplementary Figures**


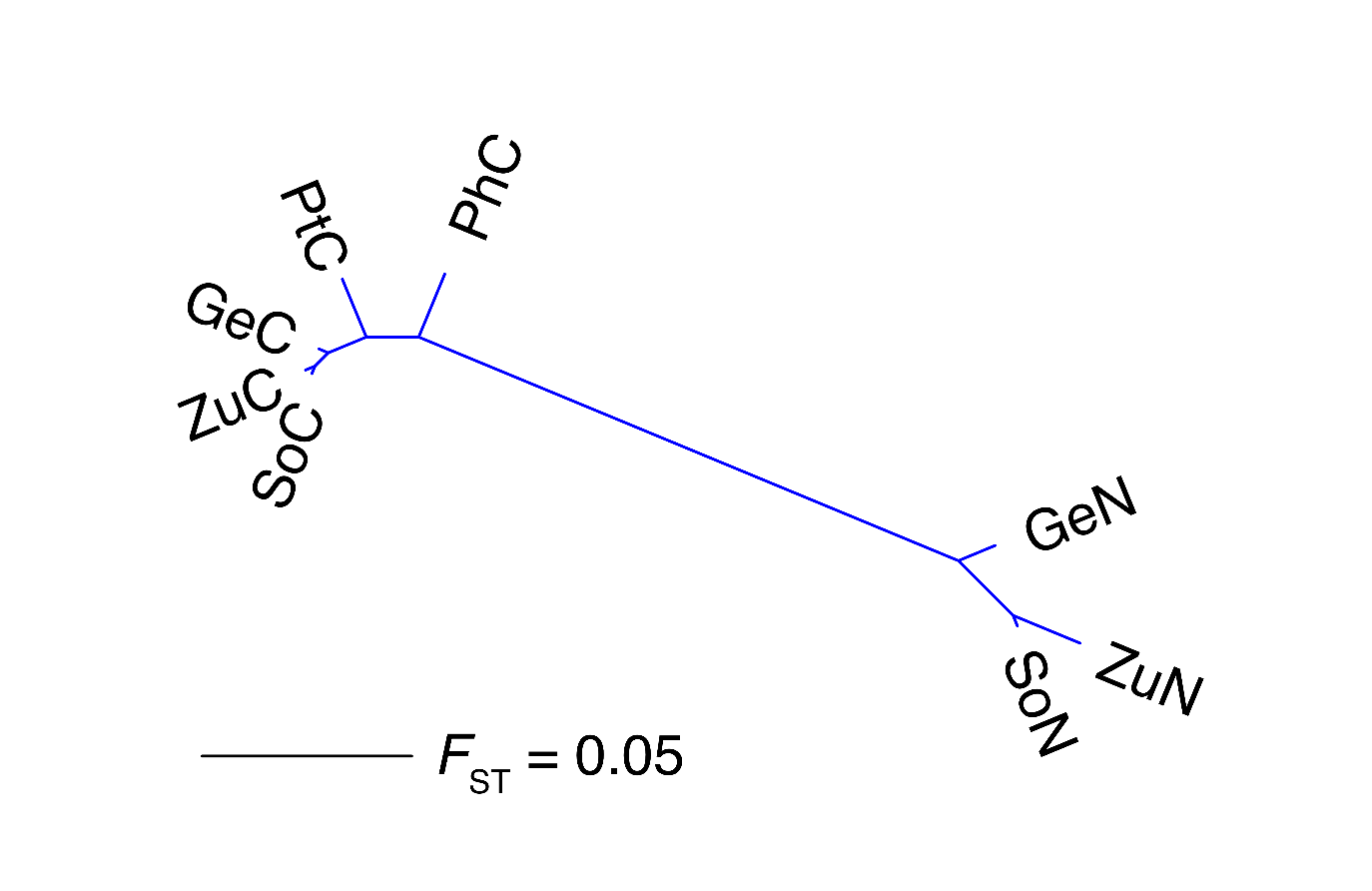


**Supplementary Figure S1.** Neighbour-joining tree of European *S. cynipsea* and *S. neocynipsea* populations based on pairwise *F*_ST_ computed from allele frequencies at 9 microsatellite loci previously obtained by Baur et al. (2020)


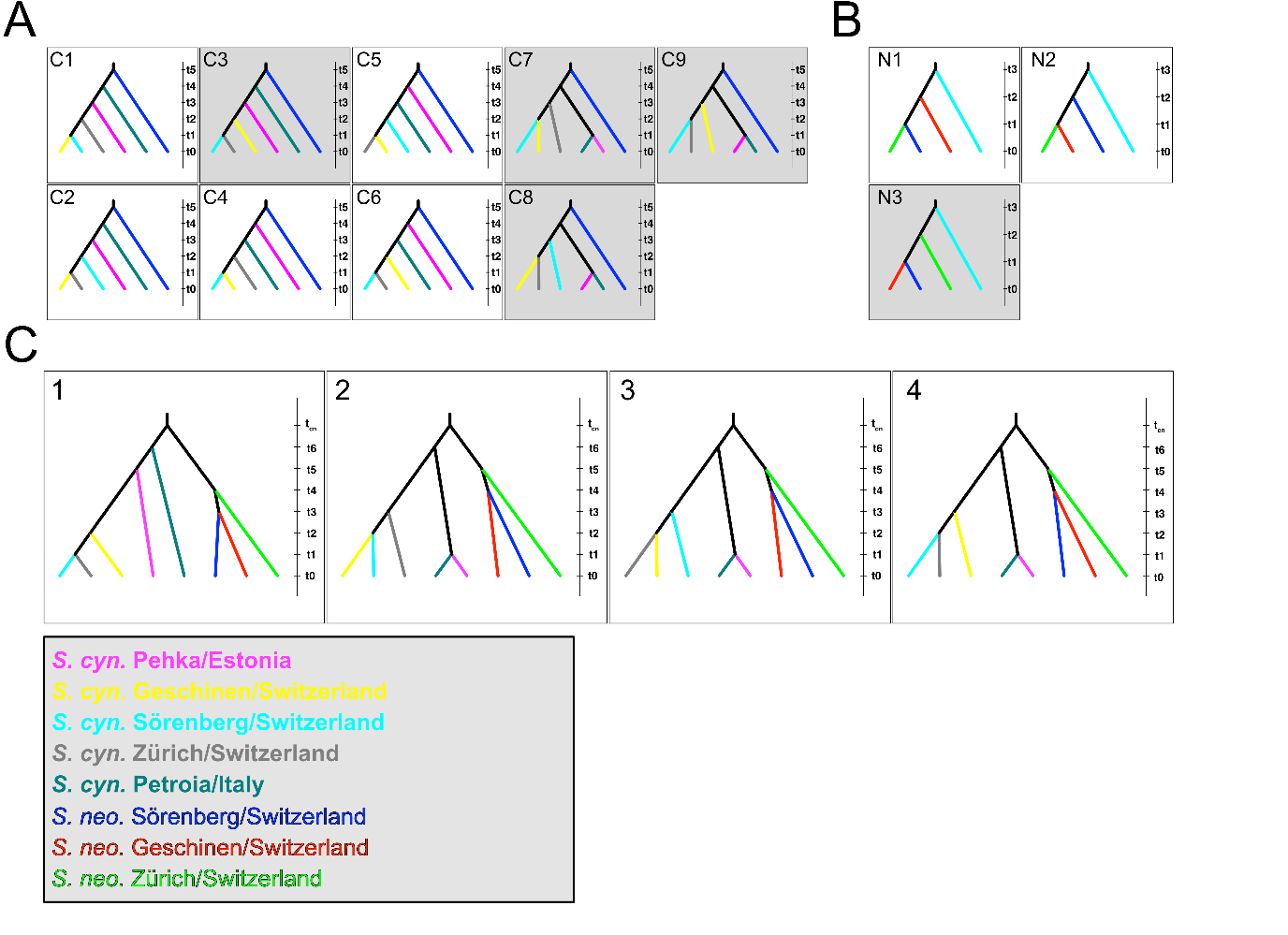


**Supplementary Figure S2.** Demographic scenarios for the evolutionary history of *S. cynipsea* and *S. neocynipsea* in Europe explored in two rounds of ABC model comparisons. (A) Nine species-specific scenarios explored in round 1 for the relationships among *S. cynipsea* populations (Scenarios C1 to C9). (B) Three species-scenarios explored in round 1 for the relationships among *S. neocynipsea* populations (Scenarios N1 to N3). The best-supported scenarios in round 1 (*cf.* Supplementary Text S3; Supplementary Table S2) are highlighted by a grey background in (A) and (B). These best-supported species-specific scenarios were combined to form all four possible demographic scenarios (scenarios 1 to 4) for the joint history of both species (C). These four joint scenarios were compared in our round 2 of ABC model comparison (*cf.* Supplementary Text S3). In all panels, $t_{cn}$ refers to the split time between *S. cynipsea* and *S. neocynipsea*. The population split times ($t_{i}$) along the y-axis are not drawn to scale.


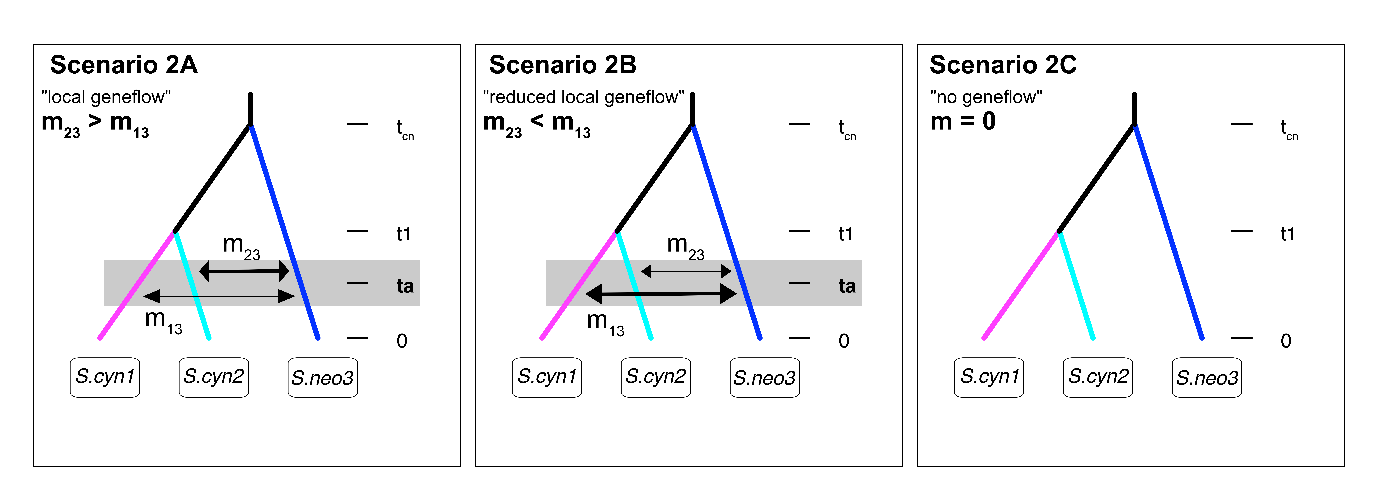
**Supplementary Figure S3.** Demographic scenarios contrasting the presence and absence of interspecific gene flow between *S. cynipsea* and *S. neocynipsea* in sympatry (parametrized by *m*_23_) vs. allopatry (*m*_13_). Scenarios shown here were derived from the best-performing full scenario (Scenario 2 in Supplementary Figure S2C) and include the three ingroups (P1, P2, P3) used in the respective ABBA-BABA test for gene flow (P4 outgroup not shown; see Supplementary Text S3). Population *S.neo3* (P3) represents any one population from the *S. neocynipsea* (right-hand) basal branch of the population genealogy in Scenario 3 (Supplementary Figure S2C), i.e. either GeN, SoN, or ZuN. Population *S.cyn2* (P2) represents the respective *S. cynipsea* population sympatric to *S.neo3*, i.e. GeC, SoC, or ZuC. Population *S.cyn1* (P1) represents any one of the two *S. cynipsea* populations allopatric to *S.neo3*, i.e. either PhC or PtC. Scenarios 2A (“local gene flow”) and 2B (“reduced local geneflow”) include bidirectional gene flow (two single pulses of bidirectional admixture) between P1 (*S.cyn1*) and P3 (*S.neo3*), and between P2 (*S.cyn2*) and P3 (*S.neo3*), at rates $m_{1\leftrightarrow3}$ and $m_{2\leftrightarrow3}$, respectively. Scenario 2C.(”no geneflow”) assumes absence of gene flow ($m_{1\leftrightarrow3}=m_{2\leftrightarrow3}=0$) and is nested within Scenarios 2A and 2B. The parameter *t*_cn_ refers to the split time between *S. cynipsea* and *S. neocynipsea*. The population split times ($t_{i}$) and the time of admixture ($t_{a}$) along the y-axis are not drawn to scale.

**Supplementary Figures S4:** Histograms of all 16 ABBA-BABA analyses showing the distribution of window-wise *D*-values in windows of 500 consecutive SNPs based on the methods of Soraggi et al. (2018; *D*_S_; left panel) and Durand et al. (2011; *D*_D_; right panel). The genome-wide average is shown as a vertical blue line and *D* = 0 is indicated by a dashed red line. Note that the x-axes may differ in scale. **All subfigures of Figure S4 can be found in the zipped folder SupplementaryFigureS4.zip.**

**
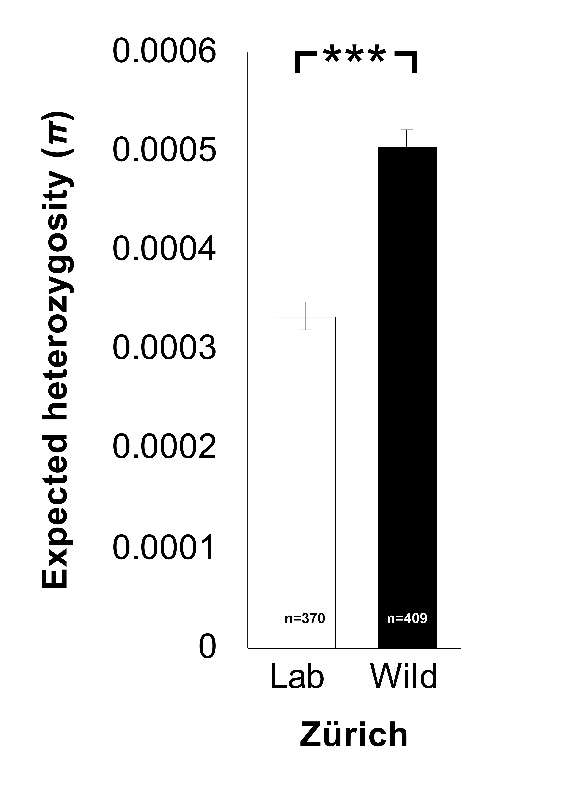
**

**Supplementary Figure S5:** Expected heterozygosity (*π*) ± SE per SNP in laboratory (Lab) after at least 30 generations of breeding vs. field-caught *S. cynipsea* samples from the Zürich site (Nature). Expected heterozygosity was estimated in non-overlapping sliding windows of 200 kb along the genome using corrections for pooled resequencing (Futschik & Schlötterer 2010) as implemented in *PoolGen* (<https://github.com/capoony/DrosEU_pipeline>; Kapun *et al.* 2020). The number of 200-kb windows analyzed are shown at the bottom of each bar. The difference in heterozygosity is significant (Mann Whitney U test, *U* = 55558, *p* < 0.001)

**Supplementary Figure S6:** Dynamics of the expected heterozygosity *π_t_* after a founder event and subsequent breeding in the laboratory over *t* generations according to Eq. S1. The dynamics are shown for a single founding female *S. cynipsea* fly mated with *N_m_* males in nature prior to being used to initiate a laboratory population of constant effective size *N_e_*. Horizontal black lines indicate the estimated expected heterozygosity in the natural *S. cynipsea* population at the Zürich site [*π*_0_, top line] and in the derived laboratory population [*π*_30_, bottom line] at generation *t* = 30 (vertical black line). Values of *π*_0_ and *π*_30_ corresponding to the two horizontal dashed lines are taken from Supplementary Figure S5. Combinations of *N_m_* and *N_e_* yielding dynamics of *π_t_* that intersects with the lower black line at generation 30 can plausibly explain the observed data. Here, this is the case for the parameter combination represented by the orange upward-pointing triangles (*N_m_* = 1, *N_e_* = 100) and the red squares (*N_m_* = 100, *N_e_* = 50), but not for the other parameter combinations.


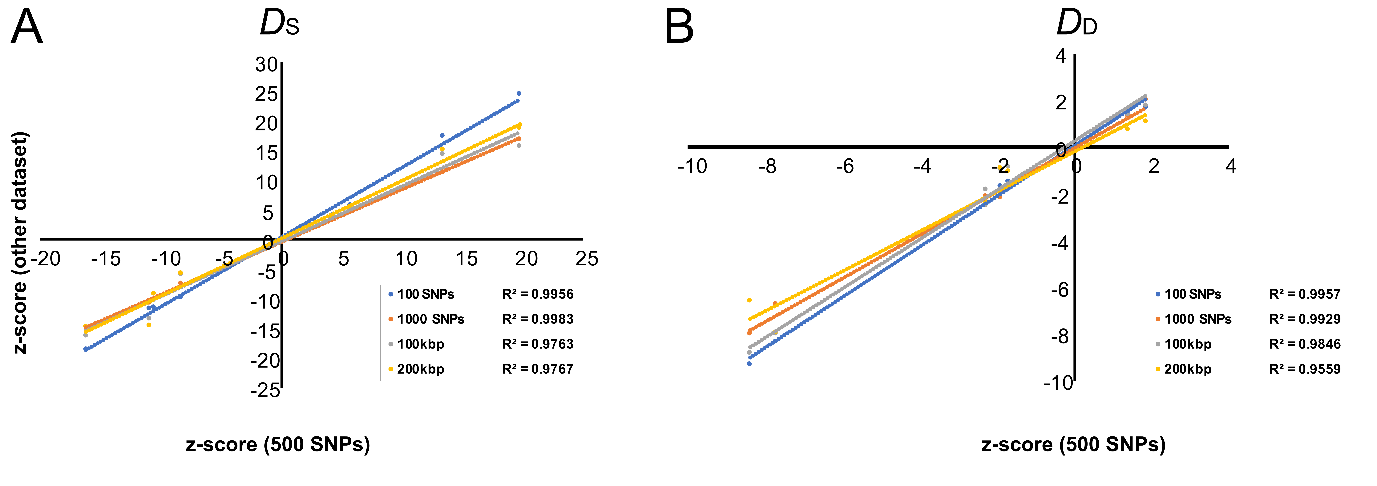


**Supplementary Figure S7:** Correlations between the *z*-scores of our chosen ABBA-BABA reference window size of 500 consecutive SNPs (on the *x* axis) with those of various alternative genomic window sizes (100 or 1000 consecutive SNPs, 100 or 200 kb) on the *y* axis. Each (differently colored) comparison entails the *z*-scores from the 6 possible ABBA-BABA tests involving the Sörenberg and Le Mourier sympatric populations as P2 and P3, and PhC, PtC, GeN and HoN populations as P1. The coefficients of determination (*R*^2^) indicate how much of the variance is explained by the corresponding linear regression in *R*. Panels A and B show results based on *D*_S_ (Soraggi *et al.* 2018) and *D*_D_ (Durand *et al.* 2011), respectively.

**Supplementary Tables**

See **GiesenEtAl_EB_SupplementaryMaterial_Tables.xlsx**

See **BaurEtAL_2020_MicSat_Raw.xlsx** for the raw microsatellite data in Baur et al. (2020)

**Supplementary Files**

See **GiesenEtAl_EB_SupplementaryMaterial_HeaderFiles.zip** for the header files that serve as the input for DIYABC.

**References**

Baur J., Giesen A., Rohner P.T., Blanckenhorn W.U., Schäfer M.A. (2020) Exaggerated male forelegs are not more differentiated than wing morphology in two widespread sister species of black scavenger flies. Journal of Zoological Systematics & Evolutionary Research, 58: 159-173. DOI: 10.1111/jzs.12327

Busing, F.M.T.A., Meijer, E. & Leeden, R.V.D. (1999). Delete-m Jackknife for Unequal m. *Statistics and Computing* **9**: 3–8

Cornuet, J.-M., Pudlo, P., Veyssier, J., Dehne-Garcia, A., Gautier, M., Leblois, R., Estoup, A. (2014). DIYABC v2.0: A software to make approximate Bayesian computation inferences about population history using single nucleotide polymorphism, DNA sequence and microsatellite data. Bioinformatics, 30(8), 1187–1189.

Deitz, K.C., Athrey, G.A., Jawara, M., Overgaard, H.J., Matias, A. & Slotman, M.A. (2016). Genome-Wide Divergence in the West-African Malaria Vector Anopheles melas. *G3: Genes, Genomes, Genetics* **6**: 2867–2879.

Durand E.Y., Patterson N., Reich D., & Slatkin M. (2011). Testing for ancient admixture between closely related populations. *Molecular Biology and Evolution*, **28**(8): 2239–2252.

Ewens, W. J. (2004) Mathematical Population Genetics. Springer, New York NY, 2nd edition, <https://doi.org/10.1007/978-0-387-21822-9>

Goldstein, D.B., Ruiz Linares, A., Cavalli-Sforza, L.L., & Feldman, M.W. (1995). Genetic absolute dating based on microsatellites and the origin of modern humans. Proceedings of the National Academy of Sciences, 92(15), 6723–6727

Goudet, J. (2005). Hierfstat, a package for r to compute and test hierarchical F-statistics. Molecular Ecology Notes, **5**(1), 184–186. doi: 10.1111/j.1471-8286.2004.00828.x

Green R.E., Krause J., Briggs A.W., *et al.* (2010). A draft sequence of the Neanderthal genome. *Science*, **328**(5979): 710–722.

Hijmans, R.J., Cameron, S.E., Parra, J.L., Jones, P.G. & Jarvis, A. 2005. Very high resolution interpolated climate surfaces for global land areas. *Int. J. Climatol.* **25**: 1965–1978.

Jeong C., Nakagome S., Di Rienzo A. (2016). Deep History of East Asian Populations Revealed Through Genetic Analysis of the Ainu. *Genetics*, **202**: 261–272.

Korneliussen T.S., Albrechtsen A., & Nielsen R. (2014). ANGSD: analysis of next generation sequencing data. *BMC bioinformatics*, **15**(1): 356.

Nei M. (1987). *Molecular Evolutionary Genetics*. Columbia University Press.

Nei, M. (1978). Estimation of Average Heterozygosity and Genetic Distance from a Small Number of Individuals. Genetics, 89(3), 583–590

Novikova YP *et al.* (2016). Sequencing of the genus *Arabidopsis* identifies a complex history of non-bifurcating speciation and abundant trans-specific polymorphism. Nature Genetics **48**: 1077–1082.

Paradis, E., Claude, J., & Strimmer, K. (2004). APE: Analyses of Phylogenetics and Evolution in R language. Bioinformatics, **20**(2), 289–290. doi: 10.1093/bioinformatics/btg412

Rannala, B., & Mountain, J.L. (1997). Detecting immigration by using multilocus genotypes. Proceedings of the National Academy of Sciences of the United States of America, 94(17), 9197–9201.

Reich, D., N. Patterson, M. Kircher, F. Delfin, M. R. Nandineni et al., 2011 Denisova admixture and the first modern human dis- persals into Southeast Asia and Oceania. Am. J. Hum. Genet. **89**: 516–528.

Soraggi, S., Wiuf, C. & Albrechtsen, A. (2018). Powerful Inference with the D-Statistic on Low-Coverage Whole-Genome Data. *G3: Genes, Genomes, Genetics* **8**: 551–566.

Weir, B. S., & Cockerham, C. C. (1984). Estimating F-Statistics for the Analysis of Population Structure. Evolution, 38(6), 1358.
